# Supplementary figures and images for: A sensory integration account for time perception
Source: PLoS Comput Biol. 2021 Jan 29;17(1):e1008668. doi: 10.1371/journal.pcbi.1008668 (PMC7875380; doi:10.1371/journal.pcbi.1008668)

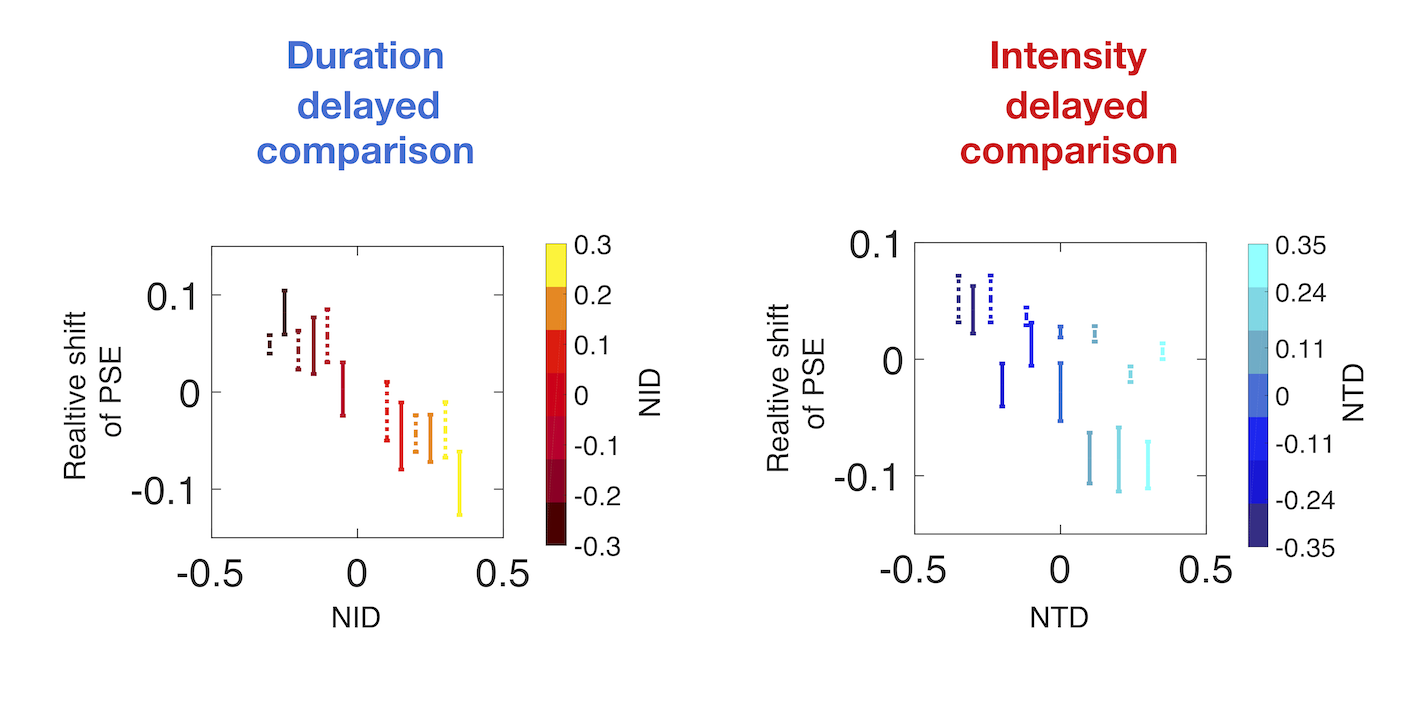

Supplement: S1 Fig — Upper panel: Duration delayed comparison task. Each bar is the standard error, centered on the mean, of the psychometric curve PSE for each NID value across all 10 human subjects (solid) and all 7 rats (dashed), relative to the PSE for the NID = 0 condition. Lower panel: Same analysis for intensity delayed comparison task. The downward slanting distribution of data indicates that, for the duration task, PSE shifted to the left as NID grew while, for the intensity task, PSE shifted to the left as NTD grew. (TIFF) [file pcbi.1008668.s001.tiff]

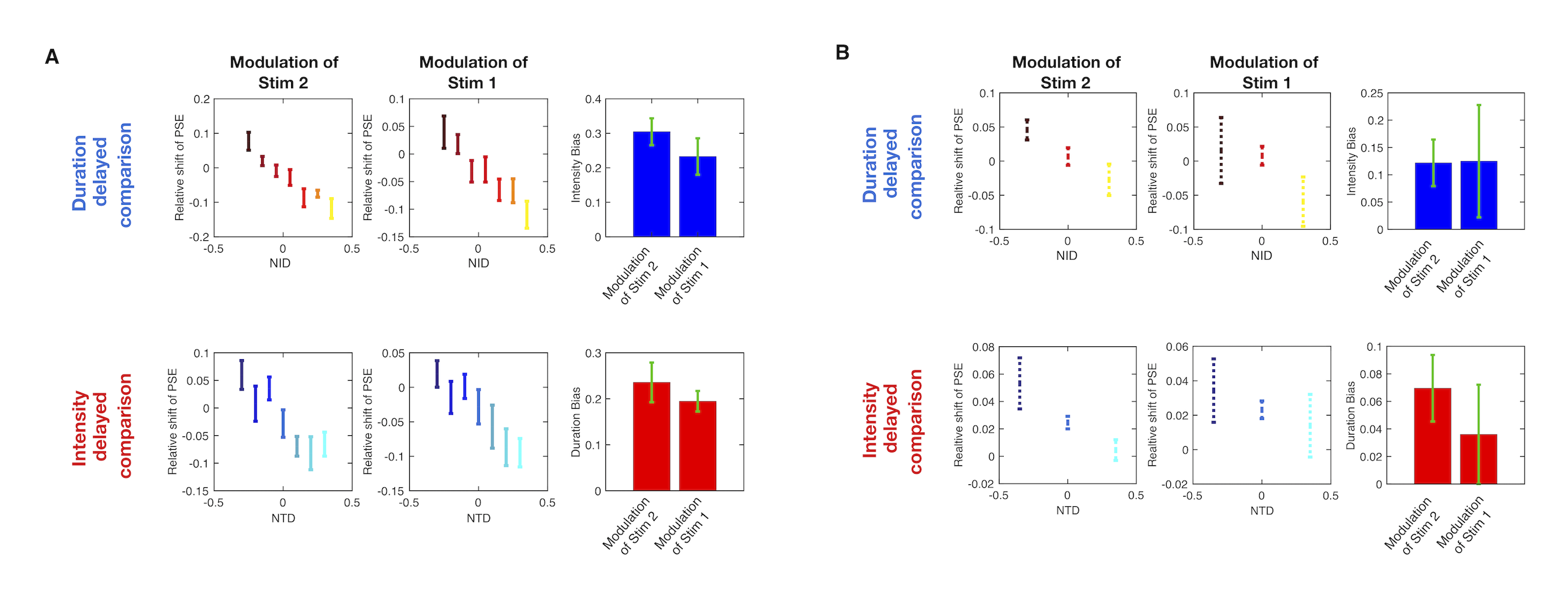

Supplement: S2 Fig — A) Upper row: Each bar is the standard error, centered on the mean, of the psychometric curve PSE for each NID value across all 10 human subjects, relative to the PSE for the NID = 0 condition, when varying the non-relevant feature of Stimulus 2 (left panel) or Stimulus 1 (middle panel), for the duration delayed comparison task. Right panel shows bias caused by the non-relevant stimulus feature, intensity, in duration comparison, for both conditions. Dots represent single subjects, while error bars represent the standard error of the mean across all subjects. Lower row: symmetrical analysis for intensity delayed comparison task. B) Same analysis as in B, for 7 rats in duration delayed comparison (upper plots), and 7 in intensity delayed comparison (lower plots). (TIFF) [file pcbi.1008668.s002.tiff]

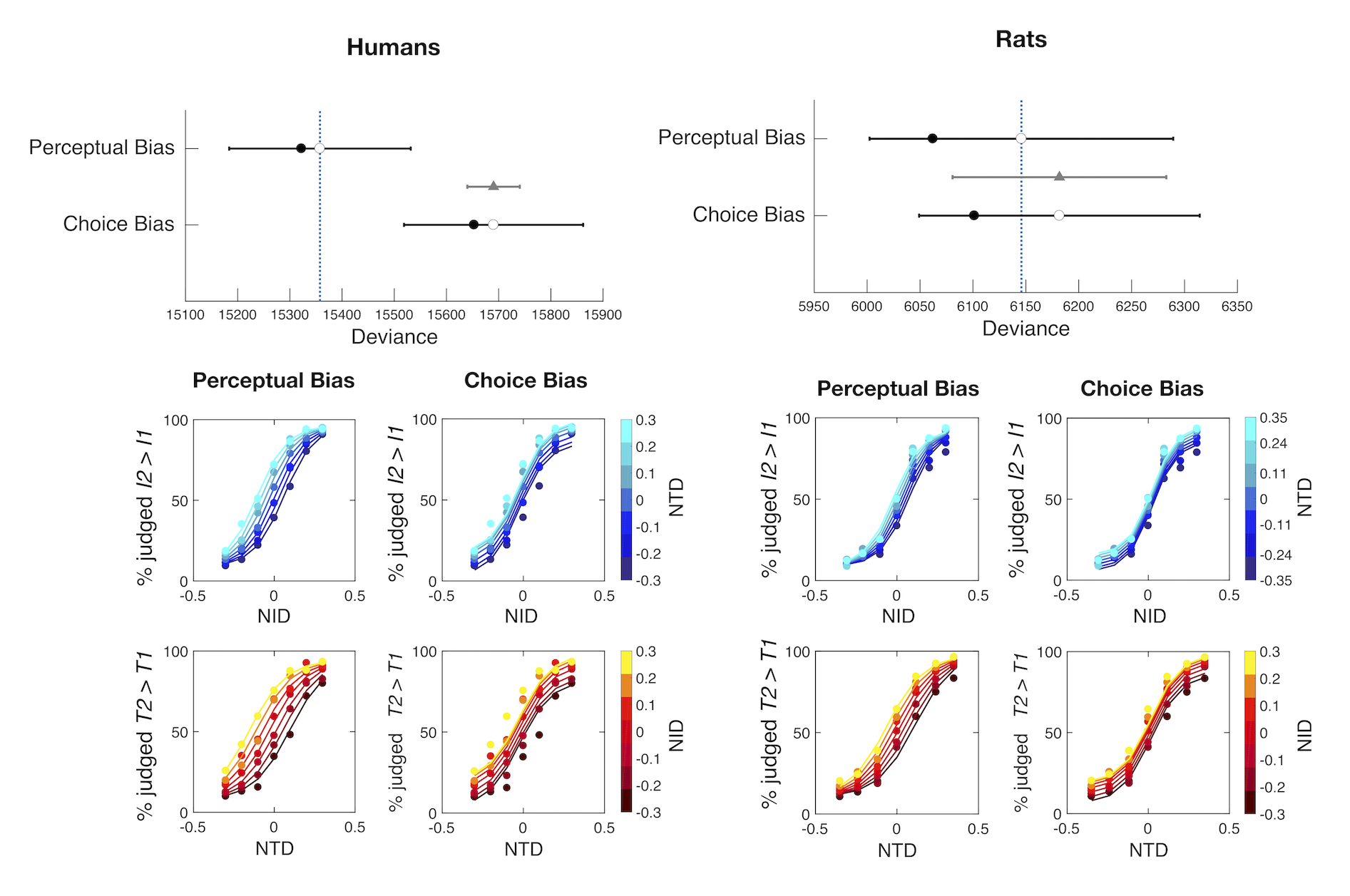

Supplement: S3 Fig — Left column shows the analysis of the rat data; right column human data. Uppermost plot shows the in and out of sample deviance estimated by WAIC using the perceptual (horizontal) and choice (vertical) biasing models. Empty dots show the out-of-sample deviance (WAIC) of each model, the filled dots show the in-sample deviance (WAIC—2 pWAIC) of each model. The black bars show the WAIC standard deviation of each model. Gray triangle shows the model’s WAIC difference, and the bar represents the WAIC difference’s standard deviation. Standard deviation is smaller, due to correlations between the computations of WAIC for each model. The middle row shows the data in the duration delayed comparison task (dashed lines) along with each model’s prediction (solid curves). The bottom row shows the data in the intensity delayed comparison task (dashed lines) along with each model’s prediction (solid curves). The columns correspond to either the perceptual or choice bias models. Much better fit is obtained in all cases by the perceptual (horizontal) shift model. (TIFF) [file pcbi.1008668.s003.tiff]

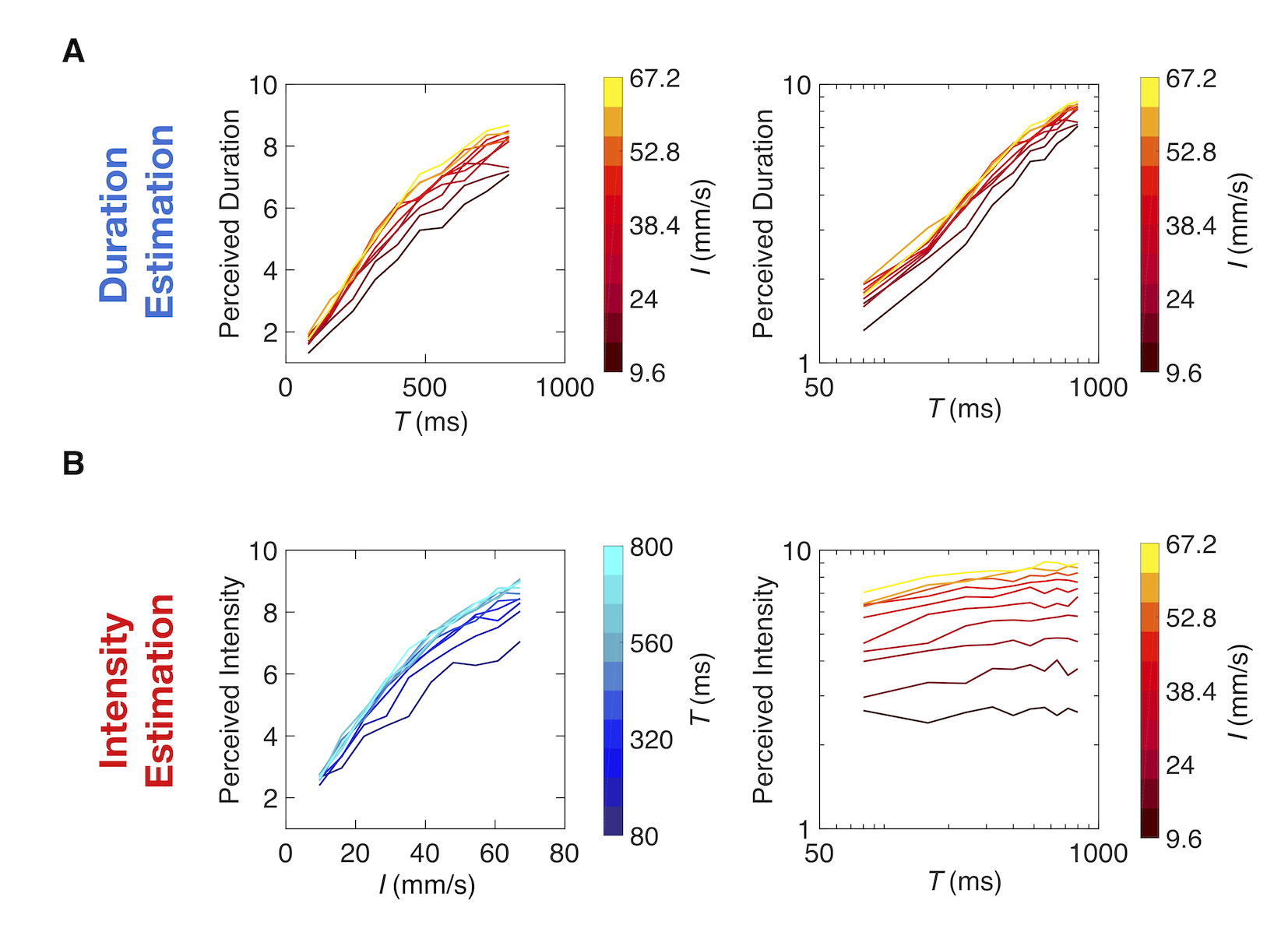

Supplement: S4 Fig — A) Duration estimation results. The two plots show the median perceived duration as a function of true duration in a linear-linear scale (left) and in a log-log scale (right), with each color denoting one I. In log scale, perceived duration increases linearly with stimulus T, suggesting a non-linear interaction between the two. B) Intensity estimation results. Left plot shows the median perceived intensity as a function of I in a linear-linear scale, with each color denoting one duration. Right plot shows the median perceived intensity as a function of stimulus T in a log-log scale, with each color denoting one I. In log scale, perceived intensity increases linearly with T, suggesting a non-linear interaction between the two. (TIFF) [file pcbi.1008668.s004.tiff]

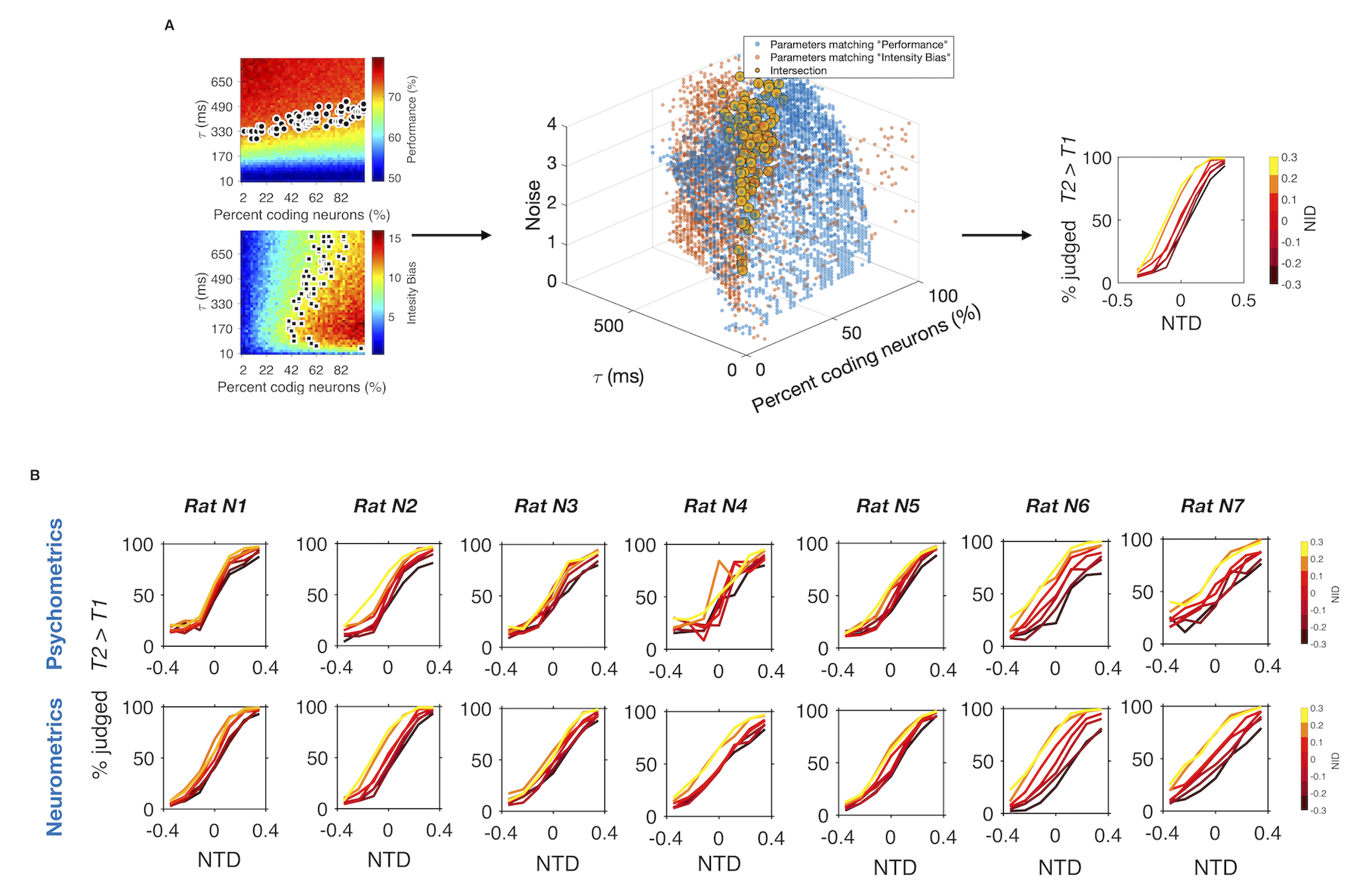

Supplement: S5 Fig — A) Leftmost plots show the leaky integrator parameters values that yielded a match of overall performance and intensity bias, for an example rat. Upper panel shows the values of τ and percent I-coding neurons that yield leaky integrator performance replicating the actual performance of the rat (black dots). Lower panel shows the values of τ and percent I-coding neurons yield leaky integrator bias replicating the intensity bias of the same rat (black dots). Middle panel shows the parameters that gave a match in performance (blue dots) and intensity bias (orange dots) in the 3d parameter space. Yellow dots indicate the parameters values that produced a match in both features. Among those parameter values, the ones that minimized the difference between the choice of the rat and the choice of the ideal observer, for all NTD and NID values, were used to generate the neurometric curves (rightmost panel). B) Psychometric curves (upper row) and neurometric curves (middle row) for all individual rats. (TIFF) [file pcbi.1008668.s005.tiff]

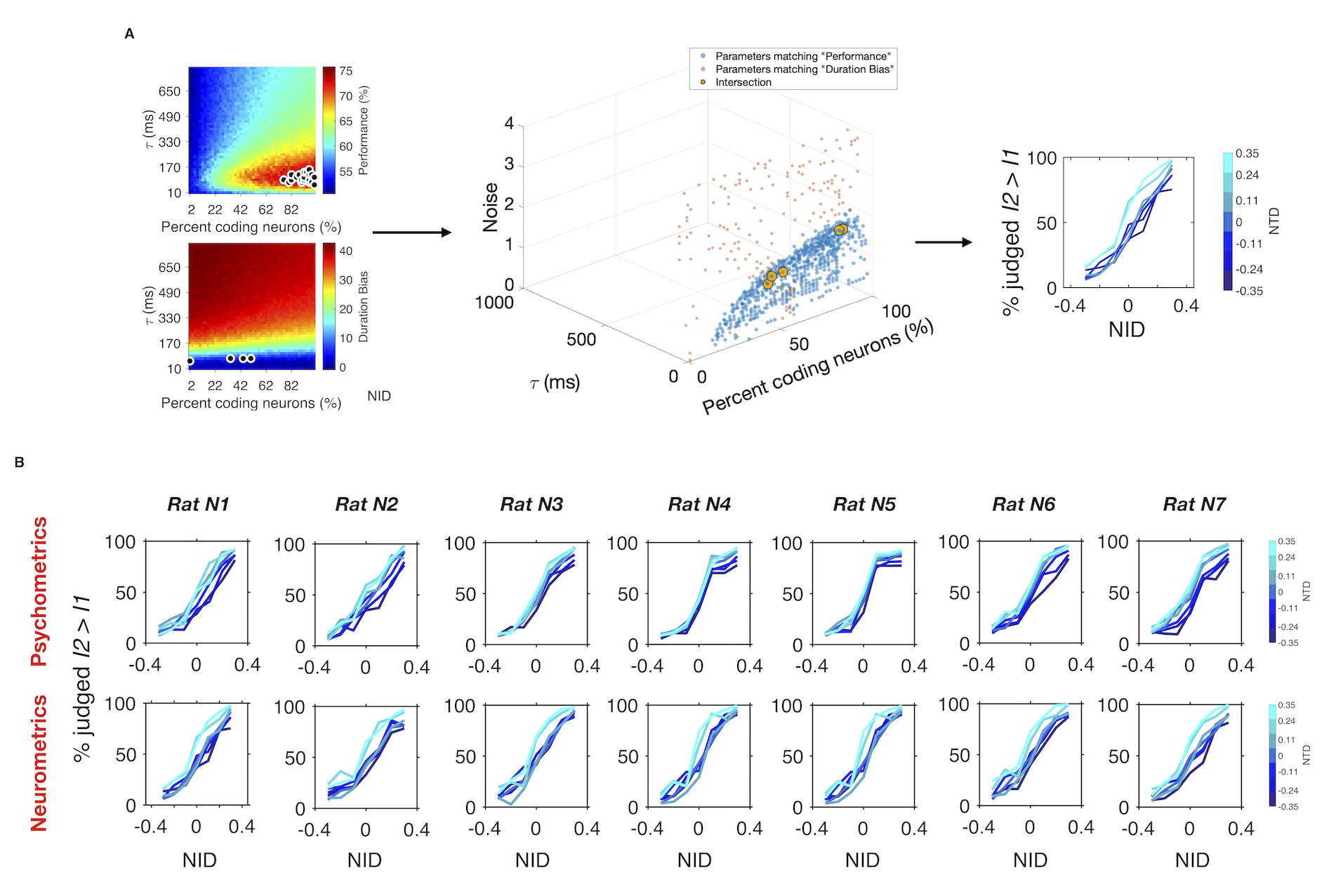

Supplement: S6 Fig — A) Leftmost plots show the leaky integrator parameters values that yielded a match of overall performance and duration bias, for an example rat. Upper panel shows the values of τ and percent I-coding neurons that yield leaky integrator performance replicating the actual performance of the rat (black dots). Lower panel shows the values of τ and percent I-coding neurons yield leaky integrator bias replicating the duration bias of the same rat (black dots). Middle panel shows the parameters that gave a match in performance (blue dots) and duration bias (orange dots) in the 3d parameter space. Yellow dots indicate the parameters values that produced a match in both features. Among those parameter values, the ones that minimized the difference between the choice of the rat and the choice of the ideal observer, for all NTD and NID values, were used to generate the neurometric curves (rightmost panel). B) Psychometric curves (upper row) and neurometric curves (middle row) for all individual rats. (TIFF) [file pcbi.1008668.s006.tiff]

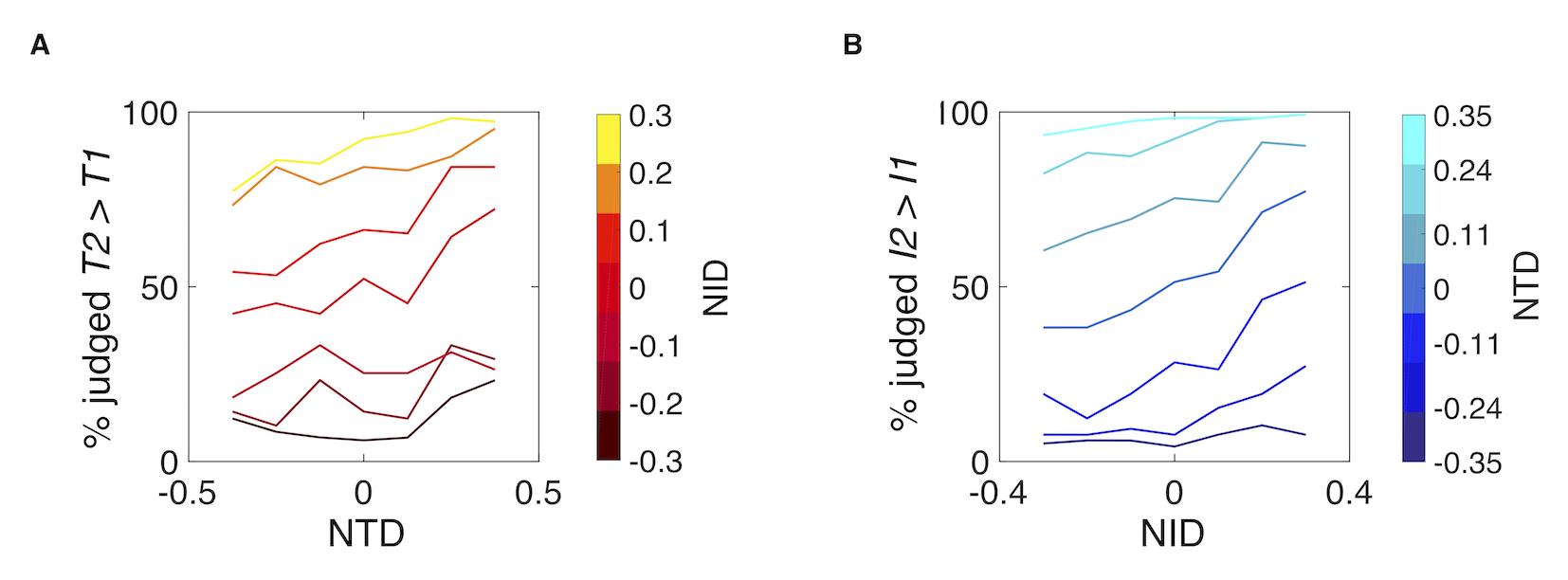

Supplement: S7 Fig — A) Neurometric curves obtained by integrating the sensory drive with the time constant suitable for the duration integrator, plotted as a function of NID values. B) Neurometric curves obtained by integrating the sensory drive with the time constant suitable for the intensity integrator, plotted as a function of NTD values. (TIFF) [file pcbi.1008668.s007.tiff]

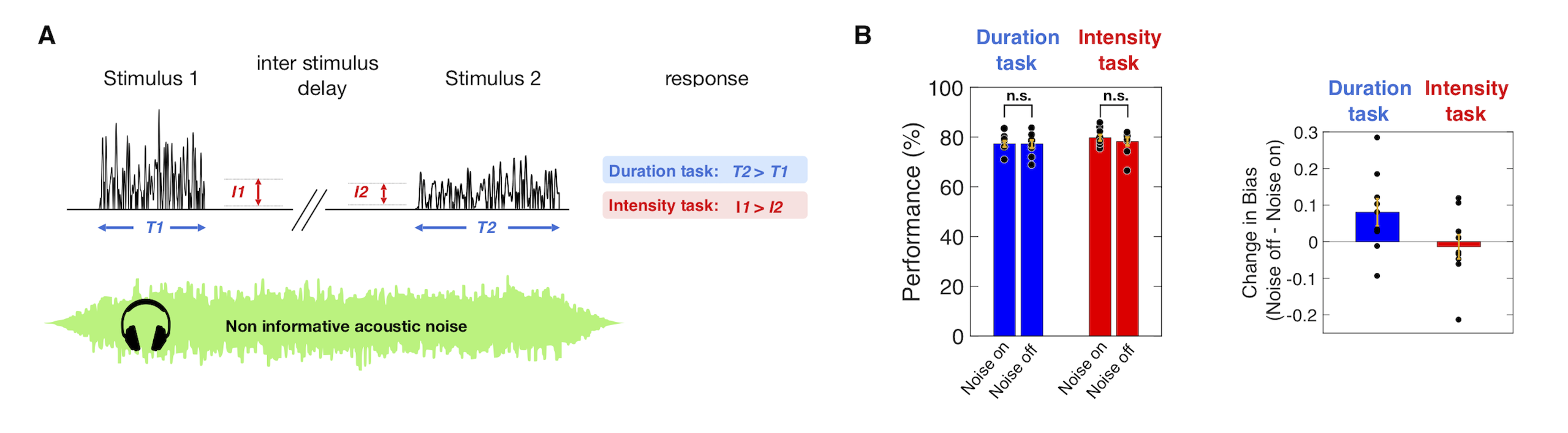

Supplement: S8 Fig — A) Schematic representation of a trial with non-informative acoustic noise delivered through headphones. B) Bars denote mean performance on the duration and intensity tasks for trials with noise on and off, across 9 human subjects. Each dot represents a single subject’s mean performance. Orange error bars are standard error of the mean across subjects. The presence or absence of noise did not affect accuracy (Kruskal-Wallis test, p = 0.72, Bayes Factor = 3.07 for the duration task, p = 0.66, Bayes Factor = 2.02 for the intensity task). C) Effect of acoustic noise on the bias caused by the task-irrelevant feature. For the duration task, the presence of noise reduced the bias normally caused by intensity (one sample, one-tailed Wilcoxon signed rank test, p = 0.0273). For the intensity task, noise did not affect the bias caused by duration (one sample, one-tailed Wilcoxon signed rank test, p = 0.5). Each dot represents a single subject’s bias difference, whilst the bar represents the average across subjects. Error bars are standard error of the mean across subjects. (TIFF) [file pcbi.1008668.s008.tiff]

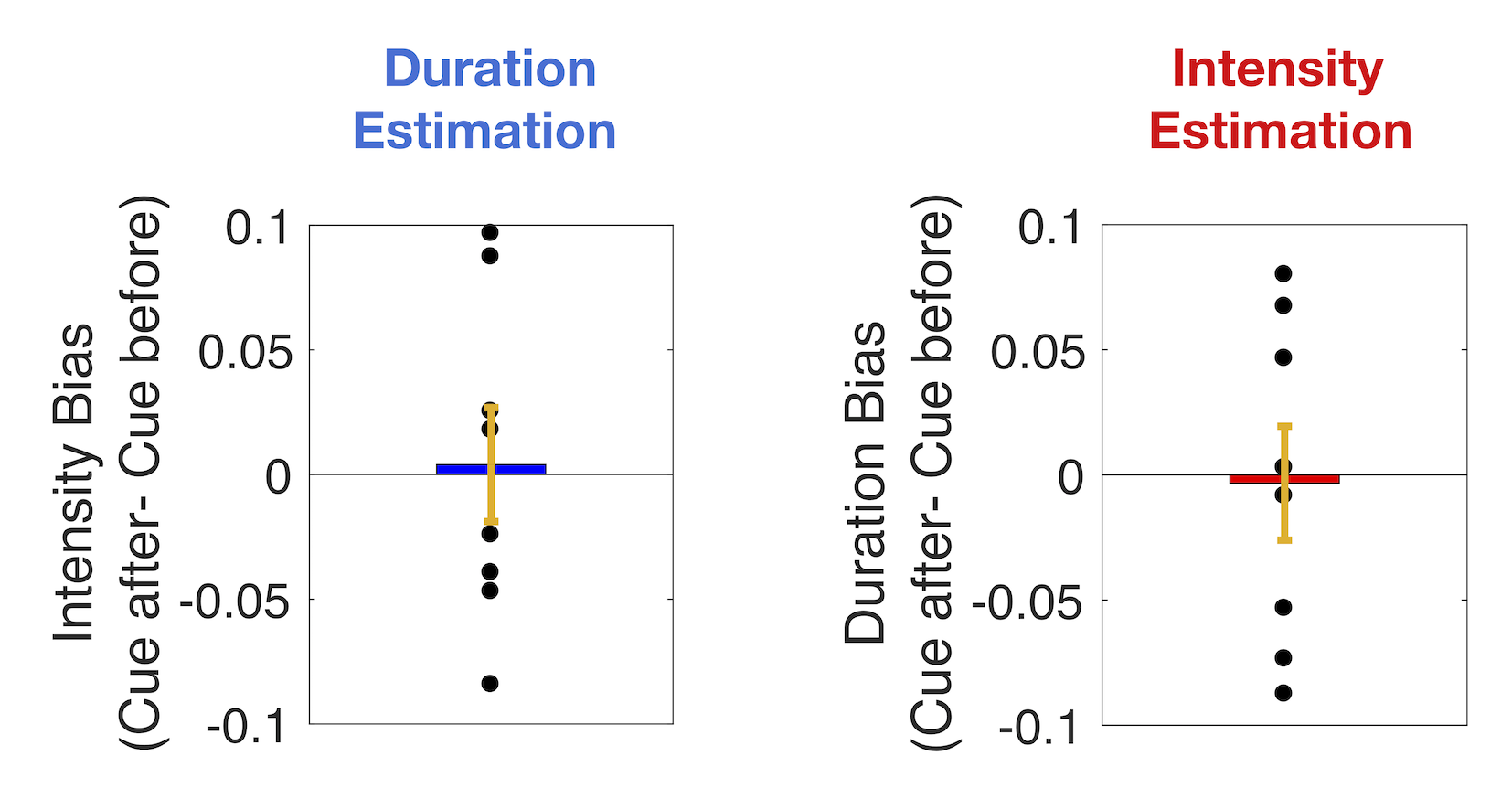

Supplement: S9 Fig — Left plot: Bias caused by the irrelevant feature (I) in the duration estimation task. Each dot corresponds to a single subject, while the bar is the mean across subjects. Right plot: Bias caused by the irrelevant feature (T) in the intensity estimation task. Each dot corresponds to a single subject; the bar is the mean across all 8 subjects. In both plots, orange error bars are standard error of the mean across subjects. Cue delivery did not affect the bias of the non-relevant feature (Kruskal Wallis test: for duration estimation p = 0.83, Bayes Factor = 2.92; for intensity estimation p = 0.75, Bayes Factor = 2.88). (TIFF) [file pcbi.1008668.s009.tiff]
