# Supplementary material for: A sensory integration account for time perception
Source: PLoS Comput Biol. 2021 Jan 29;17(1):e1008668. doi: 10.1371/journal.pcbi.1008668 (PMC7875380; doi:10.1371/journal.pcbi.1008668)
Supplement: S1 Text — (PDF) [file pcbi.1008668.s010.pdf]

Discrimination task with supplementary auditory noise

RESULTS

Fig 6G showed that, in order to reproduce the behavioral results, the duration leaky integrator needs to integrate a lower proportion of *I*-coding neurons with a higher amount of neuronal noise, as compared to the intensity leaky integrator. One possible interpretation of this result is that the duration leaky integrator integrates input from sensory areas beyond vS1. In our simulation, we can replicate the non-vS1 input by including non-*I*-coding neurons. This hypothesis is motivated by the fact that time perception is a supramodal process; all sensory channels are connected with the same sense of time [1], and the durations of multimodal (audio-visual) stimuli are known to be perceived as longer than unimodal ones [2].

We asked whether the hypothesis that the duration percept is generated by integration of sensory channels beyond those of the tactile stimulus itself could be generalized to humans. As a test, human subjects again performed the delayed comparison task; in half the trials, the tactile stimuli to be judged were accompanied by non-informative acoustic noise played through headphones (S8A Fig). Each session tested one percept, either intensity or duration.

If the irrelevant acoustic noise is integrated by both the duration and intensity leaky integrators, it will "dilute," the influence of *I*-coding neurons. In the intensity delayed comparison task this would result in a decrease in performance while in the duration delayed comparison task this would result in a reduction of the *I*-dependent bias.

The presence or absence of noise did not affect accuracy in either task (S8B Fig; Kruskal-Wallis test, $p = 0.72$ Bayes Factor = 3.07 for the duration task, $p = 0.66$, Bayes Factor = 2.02 for the intensity task). To quantify the bias induced by the non-relevant feature on the perception of the relevant

feature, as before we measured the shift of the PSE of psychometric curves caused by the non-relevant feature (see Methods). S8C Fig, shows that in the duration comparison task, the bias induced by I was significantly different between the "noise on" and "noise off" conditions (one sample, one-tailed Wilcoxon signed rank test, $p = 0.0273$), whereas in the intensity comparison task the bias induced by duration was *not* significantly different between the "noise on" and "noise off" conditions (one sample, one-tailed Wilcoxon signed rank test, $p = 0.5$). These results are not consistent with the findings predicted if both integrators were influenced by the non-relevant stimulus feature. Instead, they are consistent with the hypothesis that the irrelevant acoustic noise is integrated within the sensory drive of the duration leaky integrator, but not by the intensity leaky integrator.

MATERIAL AND METHODS

Stimulus generation

The acoustic noise was generated by stringing together sequential amplitude values at 10,000 samples/s, taken from a normal distribution. The signal was then filtered using a Tukey (also known as tapered cosine) window and delivered through headphones. The rising phase of acoustic noise amplitude was initiated 0.1-0.5 seconds (taken randomly) before Stimulus 1 vibration onset, and the falling phase occurred at a random time interval between 0.1 and 0.5 seconds, after the conclusion of Stimulus 2; in this manner, overall duration of the acoustic stimulus changed on each "*Noise on*" trial and provided no information about the vibration duration or intensity.

Experimental design

Each subject went through both an intensity and a duration delayed comparison session, in different days. The trial structure was the same as the previous delayed comparison tasks, except for the fact that in half the trials, the tactile stimuli to be judged were accompanied by non-informative acoustic

noise played through headphones. Moreover, no visual feedback was presented to subjects after their response. A total of 1,020 trials were presented at each session.

Analysis of data from delayed comparison task with supplementary auditory noise

We first characterized the behavior by using the same procedure as in the purely tactile delayed comparison task. For the intensity delayed comparison task, we then computed a linear correlation between the PSE values fitted for different NTD values, and the actual NTD values. The additive inverse of the regression coefficient, was defined as *duration bias*. Symmetrically for the duration delayed comparison task, we computed a linear correlation between the PSE values fitted for different NID values, and the actual NID values. The additive inverse of the regression coefficient, was defined as *intensity bias*.

REFERENCES

1. Kanai R, Lloyd H, Bueti D, Walsh V. Modality-independent role of the primary auditory cortex in time estimation. Exp Brain Res. 2011;209: 465–471. doi:10.1007/s00221-011-2577-3
2. De La Rosa MD, Bausenhart KM. Multimodal Integration of Interval Duration: Temporal Ventriloquism or Changes in Pacemaker Rate? Timing Time Percept. 2013. doi:10.1163/22134468-00002015
