# Supplementary material for: A sensory integration account for time perception
Source: PLoS Comput Biol. 2021 Jan 29;17(1):e1008668. doi: 10.1371/journal.pcbi.1008668 (PMC7875380; doi:10.1371/journal.pcbi.1008668)
Supplement: Attachment — Submitted filename: Reply to Reviewers.pdf [file pcbi.1008668.s011.pdf]

International School for Advanced Studies
Via Bonomea, 265
34136 Trieste, Italy

REVIEWER #1:

Reviewer #1:
Point n. 1
The MS relate two experiments and two tests of a computational model. The first experiment is tested in both rats and humans, the second consists in human testing and the computational tests ("experiment 3 and 4") based on leaky integrators.

First, I will restrain from commenting on Experiments 3 and 4, as I do not have sufficient computational expertise to comment on the specifics pros/cons. I would thus suggest an additional Reviewer on that aspect of the work.

Overall, I find the MS poorly written and not very didactic. For instance, the authors interchangeably use "intensity" (I presume, taken as the cumulative magnitude of a signal over the duration of the stimulus) and speed (here, I presume the cumulative number of changes in the signal over the duration of the stimulus) throughout text.

Reply
The reviewer misread the definitions stated in the manuscript. "Intensity" is absolutely ***not*** the cumulative magnitude over duration. Also, "speed" is carefully defined as mean speed, not the cumulative number of changes. In the manuscript we define how a vibration is constructed (lines 115-121):

> *Each vibration was constructed by stringing together over time a sequence of velocity values, v_t, sampled from a Gaussian distribution. We consider the stimuli as speed rather than velocity since earlier work has shown that perceived intensity is mapped directly from vibration mean speed. The distribution then took the form of a folded half-Gaussian and the vibration can be considered a sequence of speed values, sp_t. A single vibration was thus defined by its nominal mean speed in mm/s, denoted sp (equivalent to the standard deviation of the Gaussian multiplied by $\sqrt{(2/\pi)}$).*

Then, we define perceived intensity (lines 121-122):

> *We consider perceived intensity to be the subjective experience related to objective intensity, or sp.*

Reviewer #1:
Point n. 2
This suggests that the authors do not have a clear computational picture in mind regarding the known relation between time, intensity, and speed. It may also be very confusing considering that speed will be regarded as a process of steady changes over a given time period which none of the stimuli currently used are in fact testing.

Reply

The authors have a clear computational picture in mind. We find no grounds for the reviewer's belief that "speed will be regarded as a process of steady changes over a given time period." We have scoured the manuscript without success for any phrase that might erroneously suggest "a process of steady changes."

Reviewer #1:

Point n. 3

With respect to the empirical outcomes of the work, there is no clear novelty as to the interpretation of the time intensity trade-offs that are well known in the field.

Reply

Both the Introduction and the Discussion of the original manuscript, through numerous citations, referred to the well-known interaction between time and intensity; we never claimed novelty as regards the discovery of such an interaction. However, there might have been room for misinterpretation of the claims of the paper. As to which of our findings are claimed to be novel and which are claimed to be confirmatory of earlier work:

1. The findings *confirm* a robust effect of intensity on the duration percept ("stronger judged as longer") in humans, as already noted in the cited literature.
2. The findings *confirm* a robust effect of duration on the intensity percept ("longer judged as stronger") in humans, as already noted in the cited literature, including our own work (1).

We hope that the reviewers will see the confirmation of earlier work not as a weakness but, in this era of the "replication crisis," as a strength.

3. While most of the earlier work is in vision or hearing, our findings demonstrate both the "stronger feels longer" and "longer feels stronger" phenomena in the tactile modality, extending the principles to a new sensory domain. This is novel.
4. The "longer feels stronger" phenomenon was discovered in rats in our group (1) while the "stronger feels longer" phenomenon has not been previously demonstrated in rats. This is novel.
5. The "stronger feels longer" and "longer feels stronger" phenomena have been previously conceived as two independent effects. Studies showing one or the other phenomenon were in separate publications by different authors and were interpreted as having different underlying mechanisms. Showing both effects in a single study using a single stimulus set (single subjects in the case of humans) is novel.
6. The present study offers direct comparison of human and rat performance, thus uncovering those components of intensity perception, duration perception, and their confound that generalize across species and thus form a basic core function. Direct cross-species comparison is novel.
7. By virtue of running intensity and duration psychophysics on the same stimulus set, we were able to discern underlying parallels between two very different kinds of perception and, from there, we were able to derive a new computational model that could account for both percepts. Generating distinct percepts by the setting of parameters of a single model is novel.
8. Continuing with this section of the study, we were able to insert the empirical neuronal firing from rat vibrissal somatosensory cortex (vS1) directly into the model, Eq. (1), line 364, in place of the sensory drive term, $f(sp_t, t)$. The implementation of this data-based model with real neurophysiological led to neurometric functions consistent with the observed rat psychometric functions. This supports our main hypothesis, the dual leaky integrator model, and provides a realistic framework to explain the confound between perceived intensity and

perceived duration. The original argument that the inextricable mixing of intensity and duration arises due to a common neural input is now based not on an abstract computational model but on actual measured brain activity. All of this is novel.

9. The parameters of integration that optimize the similarity between neurometric and psychometric functions are informative about the underlying brain mechanisms and can point the field in the direction of identifying neuronal representations that encode the actual explicit percepts. This is novel.

1. Fassihi A, Akrami A, Pulecchi F, Schönfelder V, Diamond ME Transformation of Perception from Sensory to Motor Cortex. *Curr Biol* **27**, 1585-1596.e6 (2017).

Reviewer #1:
Point n. 4
I list a couple major issues re. the empirical work below:

I see two main flaws in the experimental design:

(1) The intensity and the duration of the stimuli are confounded. It is well known that increasing intensity (and more generally magnitude of a stimulus) will increase its estimated ("felt", as the authors claim) duration in all sensory modalities.

Reply
This comment leaves the authors puzzled. Is it to be read as if it is the experimental design that confounds the intensity and the duration of the stimuli? The intensity/duration confound is not a property of the experimental design – it is a property of brain function. The core of the experimental design was to document and quantify the brain's confound and to understand it according to biologically plausible mechanisms. To do so, the stimulus set fully explored both dimensions, intensity and duration, presenting vibrations that varied both parameters in 100 different combinations. The results completely work out, for the first time, the intensity/duration interaction in rodents and demonstrate that the interaction between stimulus features and percept is almost identical in rats and humans. The analysis of rat cortical activity offers a biological account for the confound.

Reviewer #1:
Point n. 5
To name a couple relevant works for this study:

Kahneman, D., & Norman, J. (1964). The time-intensity relation in visual perception as a function of observer's task. Journal of Experimental Psychology, 68(3), 215.

Allan, L. G. (1979). The perception of time. Perception & Psychophysics, 26(5), 340-354.

Kraemer, P. J., Brown, R. W., & Randall, C. K. (1995). Signal intensity and duration estimation in rats. Behavioural Processes, 34(3), 265-268.

Kuroki, S., Yokosaka, T., & Watanabe, J. (2017). Sub-Second Temporal Integration of Vibro-Tactile Stimuli: Intervals between Adjacent, Weak, and Within-Channel Stimuli Are Underestimated. Frontiers in psychology, 8, 1295.

Ball, D. M., Arnold, D. H., & Yarrow, K. (2017). Weighted integration suggests that visual and tactile signals provide independent estimates about duration. Journal of Experimental Psychology: Human Perception and Performance, 43(5), 868.

Reply
Thank you for indicating this literature. We have carefully read these works and the new manuscript refers to those that are most relevant.

Reviewer #1:
Point n. 6
(2) A constant fixed delay provides to both humans (500 ms) and animals (2 s) supra-temporal cues for resolving duration and intensity estimation. As such, the current task does not necessarily test the coding of duration but perhaps also supratemporal characteristics of the stimulus delivery.

Reply
This is incorrect, as the stimuli were built using a Stimulus Generalization Matrix (Supplementary Figure 1), wherein neither stimulus alone provides the information necessary for a correct choice; both stimuli had to be attended to and utilized to solve the task. This design makes the fixed delay uninformative for solving the task.

Reviewer #1:
Point n. 7
Additionally:
(1) Experiment 1: the "delayed comparison" tasks employed by the authors is a typical (2-IFC) 2-AFC task in psychophysics: 2 stimulus intervals are provided to the participant who has 2 alternative forced choices.
Experiment 2: the "direction estimation" task employed by the authors is a typical Likert's scale rating task.

Reply
It is true that for historical reasons the literature offers different names for similar tasks. We developed our 2-stimulus task as an evolution of the tasks developed for primates since the 1980s in the tactile modality (2) and in other modalities (3), where the common terminology is "delayed comparison working memory" task. In order to make certain that scientists from other fields recognize the format of the task, we now write that this task may also be known as a two interval two alternative forced choice task (line 124).

As to the Likert scale rating, in every instance of the literature we can find, this is a measure where the subjects must give discretized, categorical answers along a gradient (e.g. "choose an integer value from 1 to 8…"), while our rating scale is a continuous, non-verbalized measure. We think that the Likert terminology would be misleading.

2. Hernandez, Adrian, et al. "Discrimination in the sense of flutter: new psychophysical measurements in monkeys." *Journal of Neuroscience* 17.16 (1997): 6391-6400.
3. Fuster, Joaquin M., and John P. Jervey. "Neuronal firing in the inferotemporal cortex of the monkey in a visual memory task." *Journal of Neuroscience* 2.3 (1982): 361-375.

Reviewer #1:
Point n. 8
I am not clear why the authors do not use the terminology of the field (simpler for everyone to understand the benefits and limitations of the experimental design) and make us of classic Signal Detection Theory to establish the ROC, d' and biases – considering these seem to be of prime interests to the authors' main experimental questions.

Reply
Thank you for this suggestion. We have analyzed our data with signal detection theory (SDT) and did not find major advantages over using the psychometric curve parameters associated with logistic function. For instance, *d'* turns out to be well-correlated with psychometric curve slope, and the *criterion* parameter of SDT is well-correlated with the psychometric curve's point of subjective equality (PSE). However, the usage of standard *d'* measures in our task would have two main limitations: i) the *d'* measure for a 2 interval forced choice task as a $\sqrt{2}$ enhancement of the *d'* for a yes/no detection task is questioned in the literature (4), having been shown to give a poor fit of the data, and ii) the assumption of STD models is that "stimuli that are physically identical in all respects are perceived identically. But the assumption that physical identity implies perceived identity does not necessarily hold when the two stimuli differ on dimensions other than that along which they are compared." (5). This assumption is strongly violated in our data, where the perceived magnitude of the stimuli depends on two stimulus dimensions: speed and duration (Figure 3).

Carandini and Churchland (6) discussed the advantages of analysis of psychometric data by logistic curve fitting as opposed SDT. Logistic curves, unlike SDT, allow estimates of lapse rates, which were essential in our study (Supplementary Figure 4). Overall, the field is nowadays dominated by logistic functions.

4. Yeshurun Y, Carrasco M, Maloney LT Bias and sensitivity in two-interval forced choice procedures: Tests of the difference model. *Vision Res* doi:10.1016/j.visres.2008.05.008 (2008).
5. García-Pérez MA, Alcalá-Quintana R Improving the estimation of psychometric functions in 2AFC discrimination tasks. *Front Psychol* doi:10.3389/fpsyg.2011.00096 (2011).
6. Carandini M, Churchland AK Probing perceptual decisions in rodents. *Nat Neurosci* 16, 824–831 (2013).

Reviewer #1:
Point n. 9
(2) Experiment 2: the authors solely tested humans and did not counterbalance the orientation of the scales (horizontal for intensity, vertical for duration) for whom there are known cultural biases in magnitude estimations.

Reply
It is correct that we have not yet trained rats to give graded responses along a continuous dimension. The statement on scale orientation in human experiments, however, is incorrect: orientation was fully counterbalanced, as described in the original manuscript and in the new one (Results: lines 308-311; Methods: lines 775).

Reviewer #1:
Point n. 10
The observation of a linear correlation of intensity with duration estimation within a sensory modality is not surprising.
Given this strong correlation, there is no reason to believe that the participants subjectively perceived two types of signals as suggested by the authors and the decision on one task feature may permeate to the other task feature.

Reply
There is indeed a correlation between mean speed (*sp*) and duration estimation, but it is not linear, as believed by the reviewer – non-linearity is highlighted in Figure 3 of the original submission, and Figure 4C and Supplementary Figure 5 of the new submission. Regarding the next point ("Given this strong correlation…"), the study goes to great lengths to prove that the participants perceived two distinct types of signals. While those proofs were available in the original manuscript, in the new manuscript we have reanalyzed the data and produced new plots. The most cogent is Figure 2B, which measures the difference in the subject's choices (human and rat) according to the task assigned to that subject. Had subjects perceived a single type of signal as the reviewer purports, the percentage correct would not differ according to the rule used to score a choice as correct or incorrect. Instead, subjects' performance according to the unassigned rule was about 55% correct, and that deviation from chance exactly reflects the bias imposed by the irrelevant feature. The accompanying text (lines 189-199) is:

> *Figure 2B shows the overall performance achieved by humans (left) and rats (right) in the two delayed comparison tasks. The left bar of each plot depicts the percentage of correct trials obtained when the subjects' performance is analyzed by the intensity rule (the feature to be compared is vibration speed), while the right bar depicts the correct percentage when the subjects' performance is analyzed by the duration rule (the feature to be compared is vibration duration). The upper panels show that the two species had similar performance (75-80% correct) in duration delayed comparison sessions when their choices were measured according to the duration rule. However, if their choices were measured according to the relative speeds of the stimuli, performance within the same stimulus set would remain above chance (about 55% correct). The small but significant bias according to the non-relevant stimulus feature means that in both species the higher-speed stimulus, on average, tends to be judged as longer in duration. That is, stronger feels longer.*

Further, in lines 276-278:

> *In short, the main finding of Figures 2 and 3 is that subjects (humans and rats) readily extracted the stimulus feature required by the task, be it duration or intensity, but were biased by the non-relevant feature (speed or duration, respectively).*

Reviewer #1:
Point n. 11
(3) Experiments 3 & 4 consist in a computational model, which the authors do not test with neurophysiological data but suppose to take place in sensory regions. However, such model could fit just as well with a decision model, remote from perceptual decisions. The rationale for perceptual decision needs to be better explained.

Reply
[also see Reviewer #2, point n. 3 and Reviewer #4, point n. 3]
We acknowledge that this and other reviewers did not find the purely computational model persuasive. We have eliminated the original model; in the new paper we insert the measured neuronal

firing from rat vibrissal somatosensory cortex (vS1) directly into Eq. (1) in place of the term $f(sp_t, t)$. We ask whether this data-based form of the model can generate neurometric functions consistent with the observed rat psychometric functions. If so, then the parameters of integration that optimize the similarity between neurometric and psychometric functions would be informative about the underlying brain mechanisms. The implementation of this model with real neurophysiological now supports our main hypothesis, providing a realistic framework to explain the confound between perceived intensity and perceived duration.

Reviewer #1:
Point n. 12
(4) Biological plausibility and relevance of the experimental design: by definition, tactile inputs are feedback signals when the individual (human or rat) is scanning its environment moving fingers or vibrissae over a surface. I thus wonder how the lack of movement (or the passive stimulation of the sensory receptors) may fundamentally affect the pattern of behavioral responses reported here (e.g. Krakauer et al, Neuron, 2016).

Reply
Thank you for this important observation. We are aware of the need for naturalistic behaviors in the laboratory (Krakauer, Neuron, 2017) and we fully subscribe to that strategy (7). It seems the reviewer's position is that studying sense of touch in the absence of self-movement is implausible and irrelevant. But we have argued in numerous publications (7, 8) that natural whisker-mediated perception can arise through two modes of operation, generative and receptive. In the generative mode, rats "whisk" to actively seek and palpate objects. Texture, for instance, does not exist as a tactile percept until generated by the interaction of receptors with the object surface. Keen observers of rodents will note that they also operate in the receptive mode, where they immobilize their whiskers to optimize the collection of signals from an object that is moving by its own power. Receptive mode is what the reviewer calls "passive." Detecting and assessing ground vibrations, as kangaroo rats do inside their burrows and city rats do along the subway rails, does not involve self-generated motion but rather self-generated immobility. It is reasonable to think that humans immobilize their fingertips to detect motion. When physicians gauge their patients' pulse, they immobilize their fingertips on the skin, they do not palpate. In sum, the use of vibrations represents an attempt to bring naturalistic stimuli into a controlled laboratory environment, as espoused by Krakauer (2017).

7. Diamond, M. E. & Arabzadeh, E. Whisker sensory system–From receptor to decision. *Progress in Neurobiology* 103, 28-40, doi:doi:10.1016/j.pneurobio.2012.05.013 (2013).
8. Fassihi A, Zuo Y, Diamond ME. Making sense of sensory evidence in the rat whisker system. *Current Opinion in Neurobiology 60*, 76-83. doi: 10.1016/j.conb.2019.11.012 (2020)

Reviewer #1:
Point n. 13
Misc:
- Figure 1: the authors need to clarify how the intensity of the stimuli is being controlled for (aka what are their values? Are they testing speed or intensity or both?) What do NSD and NTV stand for (they are not defined before first use)?

Reply
(i) We are not sure how to answer the query "Are they testing speed or intensity or both?" The reply to point n.1 is pertinent.

(ii) As to the stimulus values, these were given in the original manuscript and can be found in the new manuscript in Methods lines 744-746, 760-763 and are depicted in Supplementary figure 1.
(iii) *NSD* and *NTD* stand for normalized speed difference and normalized time difference, respectively, as defined at their first use in the original manuscript. In the new manuscript this is in lines 154-156.

The quantities are as follows:

$NSD = (sp2\text{-}sp1) / (sp2+sp1)$

$NTD = (T2\text{-}T1) / (T2+T1)$.

Reviewer #1:
Point n. 14
- The authors may find this work relevant for time/speed estimation in these studies:

Martin, B., Wiener, M., & van Wassenhove, V. (2017). A Bayesian perspective on accumulation in the magnitude system. Scientific reports, 7(1), 630.

Tomassini, A., Gori, M., Burr, D., Sandini, G., & Morrone, C. (2011). Perceived duration of visual and tactile stimuli depends on perceived speed. Frontiers in integrative neuroscience, 5, 51.

Reply
Thank you for these suggestions. We have included the second citation. The first is about the formation of priors and is less directly connected to our work.

REVIEWER #2:

Reviewer #2:
Point n. 1
Reviewer #2:
In this manuscript, Toso et al. employed psychophysical tests in human and rats to explore the perception of stimulus intensity and the perception of time. Overall, they found that stimuli were perceived as longer in duration when the intensity is stronger; likewise, stimuli were perceived as stronger in intensity when the duration is longer. Additionally, subjects were able to judge intensity or duration with the same performance when the instructions were given before or after each trial. These results give evidence that the two percepts were produced through two separate computations that operate in parallel. Besides that, they employ leaky integration of sensory inputs to link the experimental results with this kind of models. I think that the work is important for the field and well carried out. I have some comments and suggestions that are meant to be constructive.

1) In the discussion, the authors posit that as primary somatosensory cortex exhibit short intrinsic timescale, they are not able to integrate signals temporally. Based on the hierarchical ordering proposed in a previous work (Murray et al., Nat. Neuroscience 2014), they proposed that areas with longer intrinsic timescales should be a good candidate to carry out this computation. However, it could important to include a deeper discussion proposing which cortical areas are the best candidates to accomplish the two different integrations that give rise to the perception of duration or intensity. Based on the long timescales, frontal areas are strong candidate to create the duration percept. On the hand, parietal cortical areas, as the second somatosensory cortex, are good candidates to generate the intensity percept.

Reviewer #2:
Point n. 2
2) Related with the previous point. The authors found that a non-informative acoustic noise was integrated during the formation of duration percept (Figure 5 - *Supplementary figure 9 in the new manuscript*). These results suggest that the duration percept was created in a cortical area that process sensory inputs from several modalities. Recently, it was found that neurons in the media premotor cortex (MPC) can codify acoustic or tactile stimulus frequencies employing the same parametric code for both modalities (Vergara et al., Neuron 2016). This means that single MPC neurons can integrate and transform sensory information that are first represented in two different primary cortices (A1 or S1, Lemus et al., Neuron 2010). Based on that results, MPC is a strong candidate to be involved in generating the duration percept. I think that the authors should discuss these previous results.

Reply
We reply to the related points 1 and 2 together. In the new manuscript have strengthened the discussion of the cortical regions that are the best candidates for carrying out the functions of temporal integration, including the rodent analogue of primate MPC. We added a citation of Vergara et al., Neuron 2016.

Reviewer #2:
Point n. 3
3) The authors employed a power law relationship between the sensory input and the perceived intensity and they wrote that this is consistent with the classical work of Stevens in 1959. However, as it is a relevant part of their model, I think that the authors should discuss more extensively the biological implications of this hypothesis.

Reply
[also see Reviewer #1, point n.11 and Reviewer #4, point n. 3]
As addressed elsewhere in this letter, we acknowledge that the purely computational model was not sufficiently persuasive. Among its potential weaknesses was the implementation of Stevens law to simulate the relation between sensory input and the drive provided to the leaky integrators, as noted by the reviewer. We have eliminated the original model; in the new paper we insert the empirical neuronal firing from rat vibrissal somatosensory cortex (vS1) directly into Eq. (1) in place of the term $f(sp_t, t)$. We ask whether this data-based form of the model can generate neurometric functions consistent with the observed rat psychometric functions. If so, then the parameters of integration that optimize the similarity between neurometric and psychometric functions would be informative about the underlying brain mechanisms. The implementation of this model with real neurophysiological now supports our main hypothesis, providing a realistic framework to explain the confound between perceived intensity and perceived duration.

Reviewer #2:
Point n. 4
4) Related with point 3. In Fig. 6, they showed a high disparity among the α values calculated for each subject, from α=0.05 to α=1. These discrepancies in α values give rise to completely different power law relationship. Why do the authors believe that there is this disparity? I think that the authors should analyse and discuss with more details this unexpected result.

Reply
With the implementation of the model using real neuronal spike trains, α no longer exists. Figure 6 from the previous manuscript is not included in the new manuscript.

Reviewer #2:

Point n. 5

5) Related with points 3 and 4. Is it possible to apply another biologically plausible model that explain the psychophysics results and with less disparity in the parameters across the subjects?

Reply

In the new manuscript, a biologically plausible model acting upon real somatosensory cortical neuronal spike train generates neurometric curves that are similar to the observed behavioral psychometric curves.

REVIEWER #3:

Reviewer #3:
Point n. 1

General comments

In this study, Toso and colleagues performed a series of elegant experiments showing that perception of intensity and duration interact in vibrotactile sensation. In Experiment 1, the subjects were asked to discriminate either the intensity or duration of two sequential vibrations presented to their fingertip (humans) or whiskers (rats). In Experiment 2, human subjects reported subjective duration or intensity of single vibrotactile stimulus using visual analogue scales. In both experiments, perception of two sensory attributes (intensity and duration) interacted each other. To explain these results, the authors proposed an accumulator model for perceptual decisions and performed two additional experiments to refine the model. Experiment 3 showed that the subjects were able to judge either the intensity or duration of stimulus with the same accuracy whether the task instruction was given before or after stimulus presentation. Experiment 4 showed that task-irrelevant auditory stimulus reduced the effects of stimulus intensity on duration discrimination but not vise versa, indicating that the leaky integrator for time perception receive inputs from multiple sources.

Reply

Yes, exactly. Thank you for this summary.

Reviewer #3:
Point n. 2

In general, the manuscript is well organized and is very well written. The experiments were carefully designed and the results are clearly presented. Particularly, the detailed analysis of the model should be highly appreciated. However, the interaction between stimulus intensity and time perception has been repeatedly demonstrated in vision and audition, while the present study is novel in terms of the use of somatic sensation and rodents (but only in Experiment 1). The diverse processing for different sensory attributes tested in Experiment 3 is prevalent in the sensory systems. Given that the stimulus duration should be judged based on the entire sensory inputs while the intensity is not, the different time constants of integrators for time perception and vibratory speed are expected. Furthermore, as pointed out by the authors in the end of Discussion, the present study neither support nor reject the existing models of time perception, but instead attempted to explain their behavioral data in a single class of model for interval timing.

Reply

In reply to the comment that "the interaction between stimulus intensity and time perception has been repeatedly demonstrated in vision and audition," we note that there was among the reviewers some uncertainty about which findings in the paper are claimed to be novel and which are claimed to be confirmatory of earlier work:

10. The findings *confirm* a robust effect of intensity on the duration percept ("stronger judged as longer") in humans, as already noted in the cited literature.

11. The findings *confirm* a robust effect of duration on the intensity percept ("longer judged as stronger") in humans, as already noted in the cited literature, including our own work (1).

We hope that the reviewers will see the confirmation of earlier work not as a weakness but, in the era of the "replication crisis," as a strength.

12. While most of the earlier work is in vision or hearing, our findings demonstrate both the "stronger feels longer" and "longer feels stronger" phenomena in the tactile modality, extending the principles to a new sensory domain. This is novel.
13. The "longer feels stronger" phenomenon was discovered in rats in our group (1) while the "stronger feels longer" phenomenon has not been previously demonstrated in rats. This is novel.
14. The "stronger feels longer" and "longer feels stronger" phenomena have been previously conceived as two independent effects. Studies showing one or the other phenomenon were in separate publications by different authors and were interpreted as having different underlying mechanisms. Showing both effects in a single study using a single stimulus set (single subjects in the case of humans) is novel.
15. The present study offers direct comparison of human and rat performance, thus uncovering those components of intensity perception, duration perception, and their confound that generalize across species and thus form a basic core function. Direct cross-species comparison is novel.
16. By virtue of running intensity and duration psychophysics on the same stimulus set, we were able to discern underlying parallels between two very different kinds of perception and, from there, we were able to derive a new computational model that could account for both percepts. Generating distinct percepts by the setting of parameters of a single model is novel.
17. Continuing with this section of the study, we were able to insert the empirical neuronal firing from rat vibrissal somatosensory cortex (vS1) directly into the model, Eq. (1), in place of the sensory drive term, $f(sp_t, t)$. The implementation of this data-based model with real neurophysiological led to neurometric functions consistent with the observed rat psychometric functions. This supports our main hypothesis, the dual leaky integrator model, and provides a realistic framework to explain the confound between perceived intensity and perceived duration. All of this is novel.
18. The parameters of integration that optimize the similarity between neurometric and psychometric functions are informative about the underlying brain mechanisms and can point the field in the direction of identifying neuronal representations that encode the actual explicit percepts.

We believe that to disprove every other proposed model in one publication is an unreasonably high bar. Consider that most of the literature's models are abstract and do not include neuronal data of any kind. We have proposed one plausible model that explains, simultaneously, two effects that have never been considered together.

1. Fassihi A, Akrami A, Pulecchi F, Schönfelder V, Diamond ME Transformation of Perception from Sensory to Motor Cortex. *Curr Biol* 27, 1585-1596.e6 (2017).

Reviewer #3:
Point n. 3

Thus, the present study successfully reproduced well-known phenomena in time perception and cleverly fitted the behavioral data with an accumulator model incorporating parallel processes for different attributes of vibrotactile sensation. Although both proposed models could reliably explain

the behavioral results (Fig. 6 and Supplementary Fig. 6), there is no proof of neural representation or the dominance over the other existing models of time perception.

Reply

The new manuscript addresses this concern by implementing the model using real neuronal spike trains, in substitution of the original theoretical model. Figure 6 and Supplementary figure 6 of the previous manuscript are not included in the new manuscript.

Reviewer #3:

Point n. 4

Below I suggest several specific points that might be helpful for the authors to improve the manuscript.

Specific points

In both Experiments 3 and 4, the effects of task-irrelevant sensory attribute were evaluated by the changes in bias of psychometric curves, but no information about changes in sensitivity was provided. Because selective attention can alter perceptual sensitivity, these data should also be compared between conditions.

Reply

Experiment 3 did not contain task-irrelevant features. Regarding Experiment 4, there was no difference in performance (equivalent to the reviewer's "sensitivity") between the acoustic and non-acoustic conditions, arguing that selective attention was not affected. In the new manuscript, this experiment is now presented as supplementary material in Supplementary Text 9 and Supplementary Figure 9.

Reviewer #3:

Point n. 5

Supplementary Figure 5 shows that the intensity/duration biases in Experiment 3 varied a lot from subject to subject. Were the amounts of different biases correlated?

Reply

In the new manuscript, Supplementary figure 5 from the previous manuscript is renumbered as Supplementary Figure 10. It actually shows the difference in bias between the cue-before versus cue-after conditions, not the intensity/duration bias.

To better visualize the variability of the results, we show below some extra analyses, not included in the new manuscript, as their main results were summarized as statistical scores. In the figure below, we plot all the intensity biases and duration biases between the two conditions (cue before vs cue after), for each single subject (in grey). The average bias between subjects is shown by the black dots. While different subjects show different degrees of bias, as expected, there was no significant effect of the time of cue.

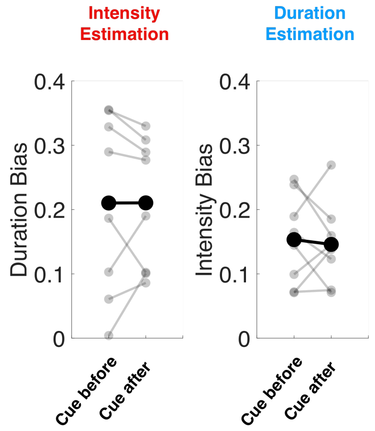

Reviewer #3:
Point n. 6
According to Model 2B, subjective passage of time would become longer in the presence of sound. It would be intriguing if the authors perform Experiment 2 with and without task-irrelevant auditory stimulus.

Reply
Model 2B from Figure 5 of the previous manuscript is not included in the new manuscript. We would like to perfrom a duration estimation task with a task-irrelevant auditory stimulus in the future. One publication cited in our manuscript (9), using a very different multimodal stimulus set from ours, suggests that we will find the effect predicted by the reviewer.

9. De La Rosa MD, Bausenhart KM Multimodal Integration of Interval Duration: Temporal Ventriloquism or Changes in Pacemaker Rate? *Timing Time Percept* doi:10.1163/22134468-00002015 (2013).

Reviewer #3:
Point n. 7
In Figure 6 and Supplementary Fig. 6, the behavioral data were fitted well with the Models 2B and 2A, respectively. It should be noted that the two models were equally reliable in this analysis.

Reply
Figure 6 and Supplementary figure 6 are not included in the new manuscript. The new manuscript addresses this concern by implementing the model using real neuronal spike trains, in substitution of the original theoretical model.

Reviewer #3:
Point n. 8
Page numbers are missing.

Reply
Apologies. The manuscript now has page numbers and line numbers.

REVIEWER #4

Reviewer #4:

Point n. 1
The manuscript by Toso et al show that more intense vibration stimuli result in longer perceived duration of stimulation, and that longer stimulation duration results in higher perceived intensity of vibration. The authors demonstrate these effects in delayed comparison tasks (using both rats and humans), as well as in a direct estimation procedure (humans). They also demonstrate that human participants can perform equivalently whether they know in advance, or only after the stimulus presentation, whether they should judge time or intensity. The authors then examine the ability of an integrator model to account for the behavioral data, and conclude that a model in which a single sensory signal is simultaneously processed by two integrators, each with their own time constant, best accounts for the data. Next, they showed that the presentation of unrelated sensory input (auditory noise) decreased the influence of vibration intensity on duration percepts, while not having an influence on the duration length bias on vibration intensity percepts. To account for this, they conclude that a downstream integration process for duration also receives input from other sensory signals, whereas the intensity integration process does not (at least with the auditory modality used). The manuscript reflects a very nice compilation of work, using multiple behavioral procedures, different species, and development/adaptation of a standard integrator model to account for the data. The authors use appropriate and sophisticated analytic techniques, the manuscript is well written, and the topic of central or distributed timing processes, as well as its relation to decision making based on accumulation of noisy sensory evidence, will be of interest to a wide audience.

Reply
Yes, exactly. Thank you for this summary.

Reviewer #4:
Point n. 2
Unfortunately, while I believe the authors' results are consistent with their interpretation that an intensity evaluation process and duration evaluation process share a common initial sensory encoding process, thereby leading to interactions between intensity and duration processing, I do not think they rule out the alternative interpretation of independent mechanisms leading to these bias.

Reply
The proposal that an intensity evaluation process and duration evaluation process share a common initial sensory encoding process is much stronger in the new manuscript. We have eliminated the original model; in the new paper we insert the empirical neuronal firing from rat vibrissal somatosensory cortex (vS1) directly into Eq. (1) in place of the term $f(sp_t, t)$. We ask whether this data-based form of the model can generate neurometric functions consistent with the observed rat psychometric functions. If so, then the parameters of integration that optimize the similarity between neurometric and psychometric functions would be informative about the underlying brain mechanisms. The implementation of this model with real neurophysiological now supports our main hypothesis, providing a realistic framework to explain the confound between perceived intensity and perceived duration.

We believe that to disprove every other proposed model in one publication is an unreasonably high bar.

Reviewer #4:
Point n. 3
[also see Reviewer #1, point n. 11 and Reviewer #2, point n. 3]

More specifically, the effect of time on intensity judgements may indeed be due to increased sampling opportunities, and the effect of intensity on timing may be due to arousal, or other similar

consequences, such as attention. Thus, just because time impacts intensity and intensity impacts time in the same experimental series (and even in the same experiment) does not necessitate that these perceptual processes share a common mechanism. Indeed, it is difficult to imagine a model in which the processes are entirely independent, as registration of the sensory stimulus must occur prior to judging its intensity or duration, and likewise it is hard to imagine a model in which time and intensity are processed completely together, since they are different properties of the stimulus. Thus, the authors' conclusions of a shared early processing stage, and independent subsequent processing stages is logically necessitated. To show that these two processes are "inextricably linked" as the authors claim in the discussion, it seems necessary to demonstrate that the judgements of time and intensity co-vary on individual trials. This would, however, require additional data…for example, using the human estimation task and asking subjects to report both the intensity and duration. Similarly, ruling out attentional or arousal based mechanisms as being responsible for the intensity biases of timing would lend support to the authors' model.

Reply

A shared early processing stage is, logically, *not* a requirement for time and intensity perception. For instance, time perception could derive from an "on" and "off" signal, where a central clock measures elapsed time between the two with no computation related to the stimulus features occurring between "on" and "off," as posited in some central clock hypotheses. It is true that the demonstration that co-variation of judgments of time and intensity on individual trials would strengthen our hypothesis. However, the experiment proposed would have some limitation in the interpretability of the results: as the report of the perceptual value is made through a motor mapping onto a slider, the hypothetical correlation between the intensity and duration perceptual reports could be due to a common motor plan. Still, Experiment 4 strongly implies just such a covariation, for the perceptual interaction was symmetric when subjects reported a single feature, even if they did not know which feature to report until the instruction cue was given after the stimulus.

In any case, we believe that the implementation of the theoretical model with spike trains recorded from rat vibrissal somatosensory cortex (vS1), reinforces our conclusion that the generation of both percepts share an early processing stage.

Reviewer #4:

Point n. 2

The conclusion that amodal bias is needed is intriguing. It would be helpful to show the full dataset in Figure 5c (i.e., as the data are presented in Figures 2 & 3), rather than a single performance variable, which can mask changes in temporal percept. Likewise, it would be helpful to specify whether participants still show a bias during noise trials, beyond showing that the bias decreases. Indeed, doesn't the model that uses the irrelevant noise as a bias, predict that duration judgements should be longer and, assuming a scalar timer, more variable?

Reply

Figure 5C of the previous manuscript is referred to as Supplementary Figure 9B in the new manuscript. The figure below - not included in the new manuscript – shows intensity biases of each subject during duration delayed comparison task, for the two conditions (noise on vs noise off). Biases decrease but do not disappear during the noise on condition.

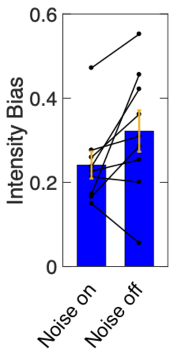

The figure below - not included in the new manuscript - shows the psychometric curves for noise on (dashed) and noise off (filled lines) conditions, obtained during duration delayed comparison task (left panel) and intensity delayed comparison task (right panel).

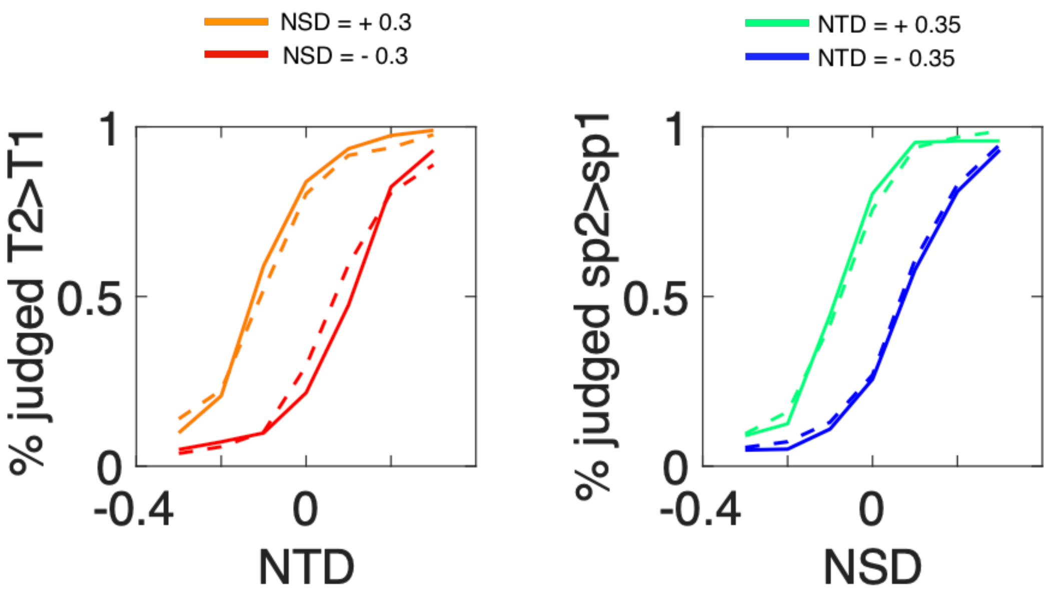

Reviewer #4:
Point n. 3
On a related point, the authors don't provide the parameter values for the bias that they conclude is necessary and use as a free parameter for fitting the human data. These should be shown. For that matter, can the best fitting parameters, and not just the ranges, be provided in Table 2?

Reply
The new manuscript addresses this concern by implementing the model using real neuronal spike trains, in substitution of the original theoretical model. Table 2 is not included in the new manuscript.

Reviewer #4:
Point n. 4
The relevant importance of alpha, tau, and the non-zero noise in the duration integration process needs to be clarified. In other words, the finding that alpha can be shared across tasks is not particularly meaningful without knowing the degree to which changes in other parameters can "make-up" for the parameter loss. For example, the authors fixed the mean of the noise parameter to zero in modelling the rat data, whereas they allowed it to vary in modelling the human data. This implies that the

parameter is of limited importance. How much better would the fits be if the noise parameter was fixed to that obtained from the human data?

Reply

The new manuscript addresses this concern by implementing the model using real neuronal spike trains, in substitution of the original theoretical model.

Reviewer #4:

Point n. 5

With regard to the data in Figure 6, can the data from individual subjects be fit with the model, and if so, which of the parameters best correlate with individual differences in accuracy, precision, or cross-task bias sensitivity?

Reply

The new manuscript addresses this concern by implementing the model using real neuronal spike trains, in substitution of the original theoretical model. Figure 6 is not included in the new manuscript.

Reviewer #4:

Point n. 6

In many of the figures, the data are restricted to a limited range on the x axis. Given the multitude of lines on each figure, it would be helpful to set the axes so that the data fill the figure.

Yellow lines/data points are very hard to see, even in the tiff images. Perhaps a light grey background would be helpful.

The amplitude of the presented noise should be provided.

The effect of intensity on duration perception has been shown in rats (Kramer et al, 1995) and pigeons (e.g., Wilkie, 1987). This work should be cited, and the sentence in the discussion about the first demonstration of these results in non-human animals should be removed.

Reply

We have reworked some of the figures to make them clearer. We changed the discussion and highlighted that one of the novelty of our animal experiment is that (lines 564-565):

> *Extracting stimulus duration and committing it to memory for future reference now enters for the first time the rodent perceptual repertoire*

This is not true for Kramer et al, 1995 and Wilkie, 1987, in which a reference memory task was used. We believe that our delayed comparison task proves that the bias of non-relevant feature on the perception of the relevant one is due to a perceptual phenomena, and not a decisional one, as showed in supplementary figure 3 and 4. This cannot be ruled out in a reference memory task.
