# Supplementary material for: A sensory integration account for time perception
Source: PLoS Comput Biol. 2021 Jan 29;17(1):e1008668. doi: 10.1371/journal.pcbi.1008668 (PMC7875380; doi:10.1371/journal.pcbi.1008668)
Supplement: Attachment — Submitted filename: Toso et al. Reply Letter.pdf [file pcbi.1008668.s012.pdf]

**Reviewer Comments to the Authors:**

**Reviewer #1, point #1**

The paper by Toso et al describes a novel computational insight into a very interesting, and puzzling, behavioral phenomenon – an intensity-duration "entanglement" (I will call it here IDE). The work integrates careful analysis of rodent and human behavior with single- and multi-unit activity from the cortex of rats performing the tasks. Having both behavioral and neuronal dynamics the authors were able to suggest a plausible model for the IDE, using dual leaky integrators with corresponding time constants for the intensity and duration integrators. they then confirmed specific predictions of the model with humans, which further strengthens its validity. Beyond providing a plausible neuronal account for the IDE phenomenon, this work establishes an excellent model for investigating neuronal mechanisms of psychophysical behaviors in general. This of course depends on the ability to achieve in rodents behaviors that are comparable to those exhibited by humans, but, as this work shows, this is feasible and it is certainly worth the effort.

This is an excellent study and I don't have any concern about its validity or value. I have, though, two suggestions that the authors may consider to use.

**Authors' reply**

Thank you for your appreciation of this work.

**Reviewer #1, point #2**

1. I was initially confused by the terminology. The authors use the term intensity to describe the mean speed. Intensity is not a quantitative term – it does not have units. Speed on the other hand is, and the authors in fact use speed units to describe the intensity variable. I suggest to seriously consider "speed" instead on "intensity" throughout the paper. I am aware to the possibility that I have missed an important consideration here, and I thus leave it to the decision of the authors.

**Authors' reply**

We now recognize that the "speed" and "intensity" terminology can be confusing. Just as physical "duration" and perceived "duration" use the same term, we have decided to reduce the potential confusion by using a single term for physical amplitude and its percept. However, we believe that "speed" will not work as the single term because human subjects (and presumably rats) would never subjectively describe the stimuli as "speedy" or "fast". That term might work for translation across the skin, as in a brushstroke, but not a vibration. Instead, we have elected to term the vibration's physical amplitude as "intensity," computed as mean speed in units of mm/s. This works because intensity has applications as both a physical parameter (e.g. sound intensity, watts/$m^2$) and a subjective experience.

**Reviewer #1, point #3**

2. a somewhat related issue. The results may indicate that there is some perceptual invariant unifying the speed and duration variables. Such an invariant may be related to the integral of speed over time for the entire duration, i.e., the distance made by the stimulus. I suggest that the authors will refer to such a possibility, possibly in light of previous relevant works, if such exist, and possibly by relevant additional analysis of their data in this direction. In any case, a discussion of this possibility is called for.

**Authors' reply**

This is an interesting point. If the actual physiological mechanism acts like a leaky integrator, then total distance travelled would not map exactly to perceived duration because the distance travelled within different time intervals would leak out to differing degrees in early versus late periods of the stimulus. However, the Reviewer's idea of a perceptual invariant is valid, in that there may be an infinite combination of durations and intensities (formerly called "speeds") that would lead to the same, invariant perceived duration. Applying our model to rats, the "iso-duration percept curves" correspond to the output $\gamma$ of the duration leaky integrator as a function of stimulus duration and intensity. REPLY LETTER FIGURE 1, below, shows the output of the model. The graph is comparable to Figure 4C, which shows the accumulated quantity over time. The output $\gamma$ ranges from blue to red (short to long), so that a given color represents the iso-duration percept. In this graph, the abscissa is stimulus intensity, and the ordinate is stimulus duration. Conceptualized as a percept, $\gamma$ does not have physical units.

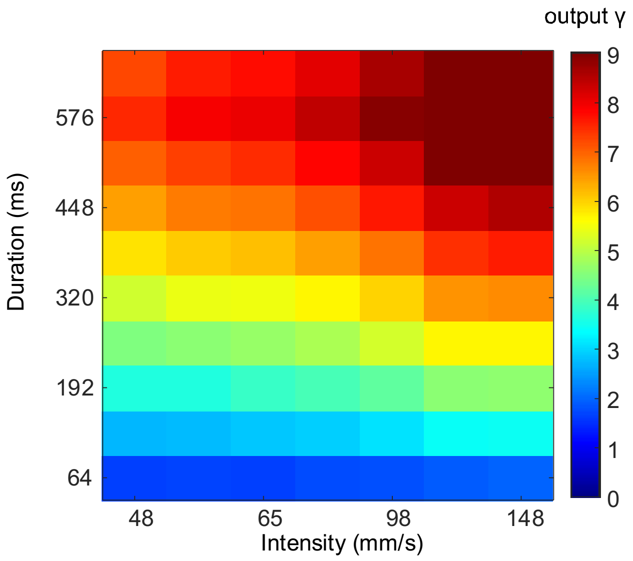


***REPLY LETTER FIGURE 1***

In humans, duration invariants are implied by the set of "slider" curves of Figure 4C. If we were to draw a dashed gray horizontal line in Figure 5C at perceived duration 6, we could derive from its intersection with yellow-to-dark red curves a sequence of actual durations ranging from about 400 to 600 ms which, once adjusted for intensity, would all be perceived as having the same duration. In REPLY LETTER FIGURE 2, below, we have replotted the data in Figure 5C with perceived duration represented by color. Perceived duration 6 is light red in this graph.

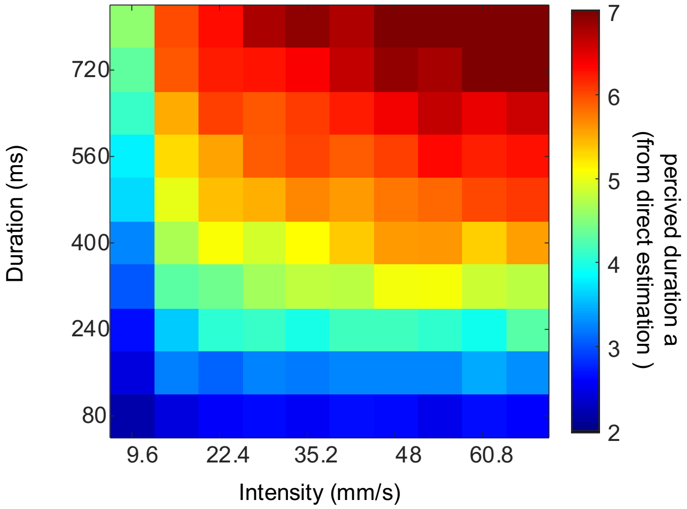


***REPLY LETTER FIGURE 2***

Although the growing size of the manuscript discourages us from including these plots in the revised document, we have discussed this very interesting point (lines 329-338):

> *The interaction between the intensity and duration implies the existence of perceptual "invariants" in humans and, presumably also in rats. Considering the set of slider curves in the left plot of Figure 5C, a horizontal line at perceived duration 6 would intersect the yellow-to-dark red curves at a sequence of actual durations ranging from about 400 to 600 ms. Once adjusted for intensity, all such stimuli would be perceived as having the same duration. Notice that the spacing in time would decrease as intensity increases, highlighting that the iso-duration percept curves would not be linear: the effect of intensity on perceived duration saturates at the upper end of the intensity scale. Similarly, the slider curves in the left plot of Figure 5D imply intensity invariants. In rats, the existence of "iso-intensity percept curves" have been previously posited in rats and humans based on the influence of vibration duration on perceived intensity (Fassihi, Akrami, Pulecchi, Schönfelder, & Diamond, 2017; Mongillo & Loewenstein, 2017).*

**Reviewer #2, point #1**

I have read with great interest the work by Toso and collagues. Generally speaking the work is quite sound (save from some issues that I will outline below), however, I have some strong doubts that this paper fits well with Plos Cmputational Biology, a prestigious journal devoted to novel aspects of neuronal computation. Let me be clear, I am not implying that the ms contains no "NEW" material. There is of course a lot of new stuff, but what is missing is that "unexpected-ness" factor. Nor the article is filling a hot gap in the litarature. Of course I may be wrong with my assessment and I ultimately leave it up to the editors but let me line out why I think this is the case.

The work first documents a mutual interference between vibration intensity and duration estimation in humans and rats. This is new but somewhat doesn't warm the reader much. Indeed the authors themselves acknowledge this in the previous editorial correspondence as well as citing much previous literature.

**Authors' reply**

Thank you for these thought-provoking observations. We point to editor Gershman's statement that journal policy does not require that a contribution contain an "unexpectedness factor" and that "strong theory-based experimental work is in my view a sufficient criterion for publication." Even if a strong "surprise factor" were obligatory for publication, we think that the paper's unexpected findings would qualify. Frequently, studies in which rodents express cognitive and psychophysical capacities rivaling those of humans have an impact on the neuroscience community. Finding the same form of confound in both species points to similar underlying mechanisms, another possibly "unexpected" factor. The 7 principal novel findings of our work were listed in earlier editorial correspondence, which the Reviewers apparently have access to.

**Reviewer #2, point #2**

The second part of the manuscript then moves onto analisys of activity in somatosensory cortex. The authors report that some neurons can code reliably vibration intensity (sp-coding), some don't. Then they hypothesize two independent read-out/accumulator mechanisms and fine tune them in order to mimic the behaviour of duration judgments and vibration intensity judgments. The result is that one need to postulate rather different mechanisms depending on which is the information that has to be extracted from the neurons. The results are quite sound, however they are not conclusive, in my view. Of course a working model is better than no model at all, however what I am missing is the fact that a computational model (just like any theory) should be able to cast some predictions. And here this part is missing.

**Authors' reply**

The working model we propose is physiologically plausible and casts many predictions, two of which are given at the end of the Discussion of the original submission and the revised submission (lines 707-713). The original text (except that "speed" has been changed to "intensity") is the following:

> *While the framework put forward in this study cannot exclude the feasibility of all other models, it does create a set of predictions that can serve to alert us as to which network properties should be sought in future physiological work. For instance, the successful generation of neurometric curves to replicate both duration and intensity perception suggests vS1 as a common input, a hypothesis that could be directly tested by optogenetic control over vS1. Our model also makes the straightforward prediction that the neuronal population implementing the readout of stimulus duration must be modulated by stimulus intensity.*

While the submitted paper does not extend to the testing of the model's predictions, it might be useful to know that we have taken the model's predictions seriously and followed up with additional studies. Two manuscripts currently in preparation demonstrate that:

(i) Optogenetic control over vS1 affects not only the rat's perceived intensity (in intensity-trained rats) but, exactly as predicted by the model, affects the rat's perceived duration (in duration-trained rats),

(ii) A brain region that has been recently proposed as the seat of time perception in rats encodes the passage of time in its population activity during judgment of vibrissal vibrations, however this region's output is *not* modulated by stimulus intensity. Our model's predictions – that the neuronal coding of duration must reflect stimulus intensity – thus allow us to disprove, or at least to present a strong piece of evidence against, the leading theory of neuronal bases of time perception.

Unfortunately, the actual tests of model prediction, listed above, amount to two full-length manuscripts in their own right, and cannot be included in the current report.

**Reviewer #2, point #3**

Finally they add another psychophysical experiment which demonstrates that the entertwinement between duration and intensity occurs even when subjects know in advance what will be the target judgment and that there is no increase in cross talk between the two dimensions if the subject is forced to hold in memory the two features and is told what to judge only after stimulus presentation. This experiment is presented as a proof of the previous model but in fact it addresses a specific independent point on the nature of this duration-intensity cross talk. In my view this feature is quite independent to the modelling work presented here. Of course I do understand that the computational framework presented here is that of a pool of encoding neurons with two independent read-out/accumulator stage, and soemwhat it is good that experiment 4 revealved the independence from working memory. Yet the results of experiment 4 could also be consistent with two entirely independent (and automatically triggered) neuronal populations. So I don't see how Experiment 4 is a proof of the model validity.

**Authors' reply**

The opening of the Introduction raises a set of questions that fit logcally with each other (lines 56-59):

> *The coupling of the perception of the content of a sensory event and the time occupied by that event raises a number of questions: Do these percepts interact with each other? Do they emerge within separate neuronal populations? Which neuronal mechanisms underlie the generation of two distinct percepts?*

The experiment referred to by the Reviewer is shown in Figure 7, where human subjects could express perceived duration or perceived intensity equally as reliably whether they received the cue about which feature to act upon either before or after stimulus presentation. Differently from what the Reviewer states, this experiment was not meant to prove or refute the leaky integrator model. Rather, it serves to answer a question posed by the earlier experiments: if the leaky integrator is accepted as a potential mechanism, should investigators expect to find a single leaky integrator with flexible integration properties set by the task, or two distinct integrators that both operate simultaneously, in parallel?

In line with the same Reviewer's desire for our study to make predictions (point #2), the cue-before versus cue-after experiment's outcome makes clear predictions – there are likely two brain regions or two distinct neuronal populations which integrate different populations of somatosensory neurons and integrate with different time constants.

**Reviewer #2, point #4**

Thus my overall feeling is that the paper is neither resolving an important quarrel in the literature, nor it is offering a compelling explanation for a realtively known phenomenon. It does offer some insights, I am not denying that but it doesn't quite pop.

This being said I will line out some specific points on the manuscript

1- Speed and Intensity are two labels chosen to represent the amount of physical vibration and its perceptual counterpart. However this choice is unfortunate. First because the two terms are lexically different. It is as if one spoke about Radiation frequency and Color (or molecular vibration and temperature). Second because outside of this paper, allude to different concepts (speed brings to mind continuous motion, not vibration - intensity brings to mind amount of energy in time). I agree that one does not need to surrender to lay terms, but some effort should be done to offer a smoother taxonomy. One option is to use "average vibration" instead od speed and "vibration intensity" for intensity.

**Authors' reply**

We have changed the labels, as explained in reply to Reviewer #1. Now, "speed" is given as "intensity" in units of mean speed, mm/s.

**Reviewer #2, point #5**

2- The prsentation of the draft leaves something to be desired. It contains inline mention of about 15 figures, yet each figure needs to be downloaded separately. More importantly some crucial aspects are deposited in supplementary material (i.e. the design matrix S1 and the swap of parameters S8) which belong much to the narrative of the paper. I don't know the space constraints of the submission but suggest to revise this. In general it would recommend to trim down the number of supplementary material to offer a better flow.

**Authors' reply**

The authors followed submission mechanisms with precision and regret the awkwardness of accessing the figures, presumably caused by journal's algorithm for compiling the final PDF. We hope that the website bugs have been corrected by the time of resubmission.

We agree that Figure S1 is crucial for the narrative of the paper. The first reason is that the stimulus design matrix constrains subjects to rely on working memory, allowing us to separate purely perceptual processes from decisional processeses. Secondly, it shows how a single set of vibration intensities and durations was used for both the *duration* and *intensity* delayed comparison tasks. This is, from our reading of the literature, a unique aspect of our work that previous studies on intensity-duration confounds did not achieve, and which allows us to build a unified framework for both perceptual features. For all these reasons have move Figure S1 into the main body where it is now Figure 2.

**Reviewer #2, point #5**

3- the paper neglects an important phenomenon (central tendency) which predicts non-linear mapping in explicit tasks (such as those of experiment 2). The issue is that with longer durations (and in general with higher noise) there is more tendency to go towards the center of the response line and this predicts a seemingly logarithmic behaviour. (see here https://www.biorxiv.org/content/10.1101/450726v1.full but also the original paper by Jazayeri et al, and follow ups by John Weadern or David Burr). This is particularly relevant here as the authors leverage on this apparent non-linearity to introduce the model (Lines 345-347). Also, I believe that assuming a later stage which bends response mappings, the parameters of the model might turn out to be different. Thus it would be good to include a reference to this point, consider the point that "non linear mapping" does not equate to "non linear encoding", and perhaps discuss how much such later non-linear stages may impact on model parameters.

**Authors' reply**

This is an important point, which we have now incorporated into the revision. The "nonlinear relations between stimulus features ($I$, $T$) and percepts" (now line 354) are clear in the data, but the Reviewer's suggestion of multiple candidate mechanisms for the nonlinearity is well taken. We fully agree that biases imposed by the history of stimuli and/or choices, such as contraction bias in working memory tasks and central tendency in estimation tasks, could exercise an effect in our task design. Other work from our lab aims to study the computational and physiological basis of such phenomena (Akrami, Kopec, Diamond, & Brody, 2018; Hachen,

Reinartz, Brasselet, Stroligo, & Diamond, 2020, under revision) and we continue to conduct experiments to investigate history biases in duration and intensity perception, in order to test the extension of our integrative model to these phenomena. These have been presented in preliminary form (e.g. Cosyne etc) and will be part of additional manuscripts. In the current manuscript, we have now stated as suggested by the Reviewer that there can be alternative causes of non linearity (lines 674-675):

> *Beyond the integrative mechanisms acting on the stimulus within the single trial, another factor that can cause nonlinearity in the percept or choice is the recent history of stimuli (37,38)*.

**Reviewer #2, point #6**

4- It wouldn't hurt if the authors attempted to cerate a verison of the model which does not exhibit the crosstalk of duration and vibration intensity. I know this is somewhat trivial but I think it would help the reader to see what's peculiar about the very parameters that enable the fit of the psychometric curves. In particular I have the impression that "short time constants for intensity" and "long time constants for duration" are somehow a general property of a good readout mechanism regardless of the cross talk. So overall I think it would be interesting to present results of the fitting procedure aslo in comparison to a model which performs unbiased estimates of duration and intensity

**Authors' reply**

As the crosstalk between duration and intensity has been consistently found in our experiments, and in the literature, we do not see what is to be gained by testing alternative models that do not predict such perceptual biases. A model of duration perception that does not exhibit the bias of stimulus intensity on perceived duration, would correspond to a leaky integrator without any intensity-coding neuron in its input. On the other hand, a model that does not show the bias of stimulus duration on percieved intensity, implies that no integration over time should take place (corresponding to a $\tau$ value much shorter than the stimulus itself), a proposed mechanism that would be at odds with many empirical findings. REPLY LETTER FIGURE 3, below, shows how the leaky integration of vS1 neurons that do not carry intensity information could lead to unbiased duration perception (left panel) and integration of intensity-coding neurons with very short time constant could lead to unbiased intensity perception (right panel). Color code is the same as that used throughout the paper.

While these forms of the model are a solid "sanity check", we do not think they increase our understanding of perceptual mechanisms because they contradict actual perceptual phenomena.

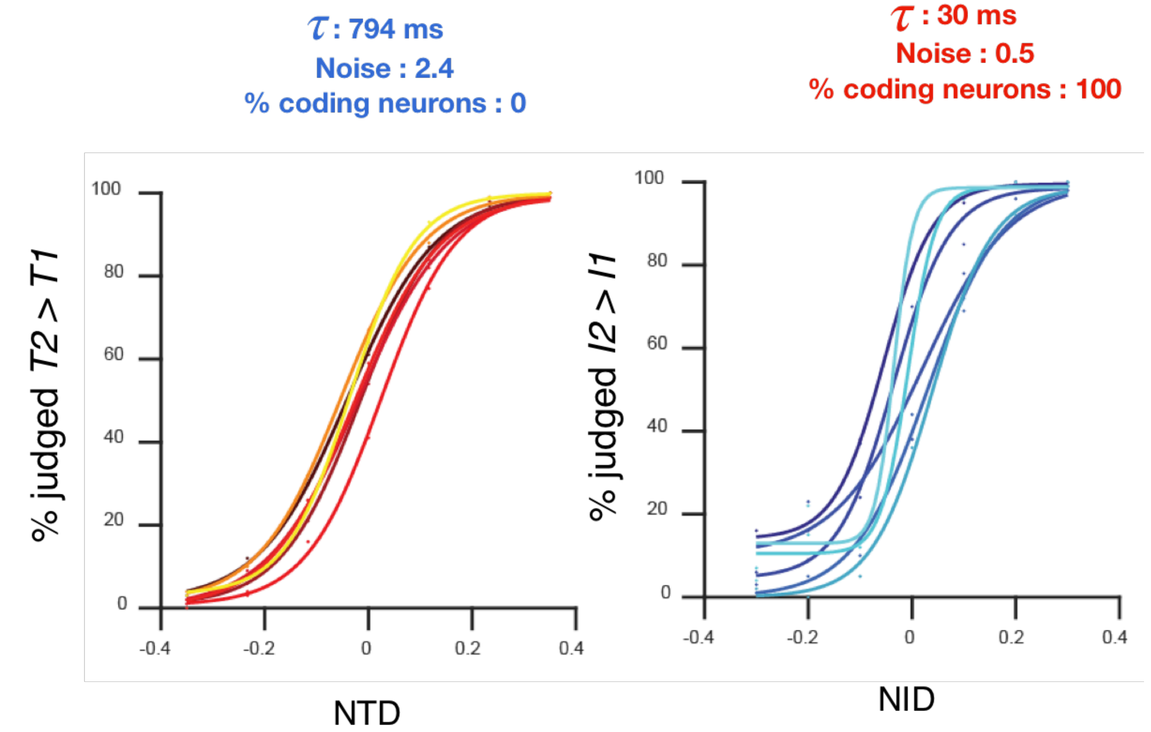


*REPLY LETTER FIGURE 3*

**Reviewer #2, point #7**

5- In general I think the paper would have gained much more relevance if the authors managed to explain also other phenomena of cross-talk such as those cited or, for instance the tau-kappa effects in vision (space-duration interference). I know this possibly means another paper, but the lack of such breadth of scope is what makes the current submission a bit too narrow in scope and, in my opinion, not up for Plsc Comp Bio

**Authors' reply**

In this work we focused on the tactile perception in humans and rodents, and were able to train 14 rats to psychophysical performance comparable to that of humans in two different behaviors. Visual tasks would go beyond the intended bounds of the present report. Still, the point of generalization beyond touch to other modalities is well taken, and we are now collaborating with two visual labs in order to extend these findings.

**Reviewer #2, point #8**

6- Figure 5G is not described in the caption. Also the result is not discussed extensively. Why would "few and noisy neurons" support duration data? And why that only in duration rats? Does this imply that traning is required in order for these neural decoders to emerge?

**Authors' reply**

We apologize for the missing Figure 5G (Now Figure 6G) caption, which occurred due to an editing error. It is now complete.

As to the difference in “Noise” and “% sp-coding neurons” parameters for duration leaky integration and intensity leaky integration, this was addressed in the manuscript (originally lines 320-333, now lines 496-508; “speed” has been changed to “intensity”):

> *The duration leaky integrator’s tolerance for non intensity-coding neurons and for noise implies that it is “open” to inputs unrelated to the vibration sensory code. This is consistent with the fact that time perception is a supramodal process; in the perceptual experience of an event, all sensory channels are connected with one unique feeling of time (12). Furthermore, multimodal (audio-visual) stimuli are perceived as longer in duration than unimodal stimuli, suggesting the convergence of separate streams (13). One possible interpretation of our data is that the duration leaky integrator normally accumulates neuronal activity from sensory areas besides vS1, reflected in the integrator’s requirement to receive non intensity-coding activity with high noise within its sensory drive. On this basis, we predicted that the percept of duration (but not intensity) could be affected by input carried within a sensory modality other than that whose duration must be judged. Psychophysical experiments in human subjects support the prediction (see Supplementary figure 8 and Supplementary text 8), revealing that an acoustic stimulus delivered together with tactile vibration is accumulated by the duration integrator but not the intensity integrator.*

As to whether training is required in order for these neural decoders to emerge – this is a great question. At the moment we do not know if the integrative properties of the neuronal populations involved in the two tasks emerge through training, or else are present even before the human or rat learns the task. In order to answer this question, the lab is working on training single animals in both *intensity* and *duration* delayed comparison tasks. By employing extracellular recordings in behaving animals from putative regions that could be involved in the integration process (vibrissal M1, M2, and surrounding frontal cortical regions), we will be able to see how the two circuits emerge during training and will assess whether the animal can make use of one or the other circuit in a flexible and task-dependent manner. If we had to speculate, we would posit that the integrative mechanisms leading to an intensity representation and a duration representation exist even before training, because such percepts are likely to be experiences in any animal’s life. We suppose that the training involves the transformation of such (innate) percepts to choices. But we do not have data available yet, unfortunately.

**Reviewer #2, point #9**

7- Whenever claims of independence are made, see for instance results of exp4 - Figure 6, Bayes factors should be employed.

**Authors’ reply**

Bayes factors have been added, together with p-values.

**Reviewer #2, point #10**

8- I am not too fond of the usage of the verb "to accord" So I suggest these changes:

Line 614- Accord -> Fit(s)

Line 641- Accordance -> Agreement

**Authors’ reply**

Changed.

*LITERATURE CITED IN THE REVIEW LETTER*

Akrami, A., Kopec, C. D., Diamond, M. E., & Brody, C. D. (2018). Posterior parietal cortex represents sensory history and mediates its effects on behaviour. *Nature*, *554*(7692), 368–372. https://doi.org/10.1038/nature25510

Fassihi, A., Akrami, A., Pulecchi, F., Schönfelder, V., & Diamond, M. E. (2017). Transformation of Perception from Sensory to Motor Cortex. *Current Biology*, *27*(11). https://doi.org/10.1016/j.cub.2017.05.011

Hachen, I., Reinartz, S., Brasselet, R., Stroligo, A., & Diamond, M. E. (2020). Dynamics of history-dependent perceptual judgment. *BioRxiv*.

Mongillo, G., & Loewenstein, Y. (2017, June 5). Neuroscience: Formation of a Percept in the Rat Cortex. *Current Biology*, Vol. 27, pp. R423–R425. https://doi.org/10.1016/j.cub.2017.04.019
